# The absence of murine cathelicidin-related antimicrobial peptide impacts host responses enhancing *Salmonella enterica* serovar Typhimurium infection

**DOI:** 10.1186/s13099-020-00386-1

**Published:** 2020-11-13

**Authors:** Danisa M. Bescucci, Sandra T. Clarke, Catherine L. J. Brown, Valerie F. Boras, Tony Montina, Richard R. E. Uwiera, G. Douglas Inglis

**Affiliations:** 1 Lethbridge Research and Development Centre, Agriculture and Agri-Food Canada, Lethbridge, AB Canada; 2 Department of Agricultural, Food and Nutritional Science, University of Alberta, Edmonton, AB Canada; 3 Department of Biological Sciences, University of Lethbridge, Lethbridge, AB Canada; 4 Chinook Regional Hospital, Alberta Health Services, Lethbridge, AB Canada; 5 Department of Chemistry and Biochemistry, University of Lethbridge, Lethbridge, AB Canada; 6 Southern Alberta Genome Sciences Centre, University of Lethbridge, Lethbridge, AB Canada

**Keywords:** Mice, Cathelicidin, mCRAMP, *Salmonella enterica* Typhimurium, Microbiota, Colonization resistance

## Abstract

**Background:**

Cathelicidins are a class of antimicrobial peptide, and the murine cathelicidin-related antimicrobial peptide (mCRAMP) has been demonstrated in vitro to impair *Salmonella enterica* serovar Typhimurium proliferation. However, the impact of mCRAMP on host responses and the microbiota following *S.* Typhimurium infection has not been determined. In this study mCRAMP^−/−^ and mCRAMP^+/+^ mice (± streptomycin) were orally inoculated with *S. enterica* serovar Typhimurium DT104 (SA +), and impacts on the host and enteric bacterial communities were temporally evaluated.

**Results:**

Higher densities of the pathogen were observed in cecal digesta and associated with mucosa in SA+/mCRAMP^−/−^ mice that were pretreated (ST+) and not pretreated (ST−) with streptomycin at 24 h post-inoculation (hpi). Both SA+/ST+/mCRAMP^−/−^ and SA+/ST−/mCRAMP^−/−^ mice were more susceptible to infection exhibiting greater histopathologic changes (e.g. epithelial injury, leukocyte infiltration, goblet cell loss) at 48 hpi. Correspondingly, immune responses in SA+/ST+/mCRAMP^–/−^ and SA+/ST−/mCRAMP^–/−^ mice were affected (e.g. *Ifnγ, Kc, Inos, Il1β, RegIIIγ*). Systemic dissemination of the pathogen was characterized by metabolomics, and the liver metabolome was affected to a greater degree in SA+/ST+/mCRAMP^–/−^ and SA+/ST−/mCRAMP^–/−^ mice (e.g. taurine, cadaverine). Treatment-specific changes to the structure of the enteric microbiota were associated with infection and mCRAMP deficiency, with a higher abundance of *Enterobacteriaceae* and *Veillonellaceae* observed in infected null mice. The microbiota of mice that were administered the antibiotic and infected with *Salmonella* was dominated by *Proteobacteria.*

**Conclusion:**

The study findings showed that the absence of mCRAMP modulated both host responses and the enteric microbiota enhancing local and systemic infection by *Salmonella* Typhimurium.

## Background

Host-defense peptides are an evolutionary conserved component of the innate immune system that play an essential role in protection of the host [1]. Antimicrobial peptides are comprised of defensins, C-type lectins, and cathelicidins [2]. Cathelicidins are peptides characterized by an N-terminal signal peptide, a cathelin-like propeptide, and a variable C-terminal domain, which is cleaved to release the antimicrobial activity [3]. These antimicrobial peptides have been identified in mammalian species including rats [4], human beings [5], rabbits [6], monkeys [7], pigs [8], and cows [9], among others. In mice, the only cathelicidin that has been identified is the murine cathelicin-related antimicrobial peptide (mCRAMP) [10]. mCRAMP was first isolated from bone marrow and has been the focus of investigation due to the homology in gene sequence, structure, and protein processing that it shares with the human cathelicidin, LL-37/hCAP-18 [11]. mCRAMP is mainly expressed in neutrophils; however, its presence has also been observed in the testis, lung, urinary tract, and gastrointestinal tract [10]. mCRAMP is an amphipathic α-helical structure that binds to negatively charged groups of the outer bacterial membrane, thus altering its structure and permeability with ensuing bactericidal activity [12]. Several studies using mCRAMP null mice have shown the role that this cathelicidin plays in the protection of skin [13], the urinary tract [14], and the gastrointestinal tract (GIT) [3]; however, the studies conducted in the GIT were mainly restricted to the colonic tissue, and targeted colitis incited by *Candida albicans, Escherichia coli* and *Citrobacter rodentium* [15, 16]. Cathelicidins also participate in modulation of immune responses, including chemoattraction of leukocytes by activation of formyl peptide receptors (FPR) [17], stimulation of degranulation [18], enhancement of phagocytosis [19], and stimulation of humoral adaptive immune responses [17]. mCRAMP has also been shown to stimulate neovascularization of cutaneous wounds [20], and to play a pivotal role in maintaining homeostasis of the colonic microbiota [21].

It is well known that the commensal microbiota confers protection to the host by competing directly and indirectly with enteric pathogens, referred to as colonization resistance [22]. The precise mechanisms by which colonization resistance functions are enigmatic at present. However, direct competition between pathogenic microorganisms and the autochthonous microbiota has been suggested, including competition for nutrients [23] and niches [24], delivery of bactericidal effectors by means of the type VI secretion system [25], and production of anti-microbial compounds such as bacteriocins [26]. Indirect mechanisms of colonization resistance include stimulation of short chain fatty acid production [27], pro-inflammatory cytokines [28], and antimicrobial peptides [15]. As a consequence, when the structure of the autochthonous microbiota is affected (i.e. reduction in diversity), the ensuing dysbiosis can result in a loss of homeostasis, including the loss of colonization resistance, with associated negative health implications for the host [29].

*Salmonella enterica* serovar Typhimurium is a Gram-negative intracellular pathogen that can incite a wide spectrum of clinical manifestations that vary from mild enterocolitis to fulminating septicemia [30]. In mice, infection by this pathogen produces a typhoid-like fever disease [31]. Disruption of the commensal microbiota in mice via administration of streptomycin before inoculation with *S.* Typhimurium has been used to mimic and thus study human salmonellosis characterized by typhlitis, colitis, and diarrhea [32, 33]. However, the temporal clearance of the microbiota is an important limitation of this model due to the disruption and suppression of the mechanisms of colonization resistance conferred by the autochthonous microbiota. In this regard, investigations have shown that mice harbouring a disrupted microbiota are highly susceptible to enteric infections by pathogens [34, 35]. The importance of the microbiota in modulating host immune responses has been extensively studied. The recognition of commensals by toll-like receptors (TLRs) is essential to maintain intestinal homeostasis [36], and recognition of bacteria by TLRs is an important process to induce antimicrobial host defense responses [37]. mCRAMP released via TLR stimulation has previously been described to impair mouse colitis [16]. However, the role that mCRAMP plays in the initiation and progression of salmonellosis in vivo, including colonization resistance against *S.* Typhimurium, is unknown.

We hypothesized that the host defense peptide, cathelicidin, aids in protecting the host from salmonellosis by modulating the intestinal microbiota and host immune responses. We comparatively and temporally evaluated the impact of cathelicidin on the enteric microbiota as well as host immune responses, including mucosal responses to *S.* Typhimurium DT104 (SA+) relative to non-infected (SA−) mice. We also ascertained the effects of cathelicidin by measuring responses in cathelicidin deficient (mCRAMP^−/−^) and cathelicidin competent (mCRAMP^+/+^) mice. Moreover, we included a streptomycin sulfate (ST+) or no streptomycin (ST−) treatment to induce a disruption of the microbiota composition [38], and to contrast responses in mice with and without a dysbiosis. Our data supported our hypothesis that the absence of mCRAMP predisposed mice to enteric and systemic salmonellosis by triggering an innate immune response and modifying the composition of the autochthonous microbiota. In addition, our evidence suggests that the use of mCRAMP knockout mice may be a suitable alternative to the conventional streptomycin dysbiosis model for studying *Salmonella* enterocolitis in mice.

## Results

### mCRAMP modulated histopathologic damage caused by *Salmonella* Typhimurium

**Fig. 1:**
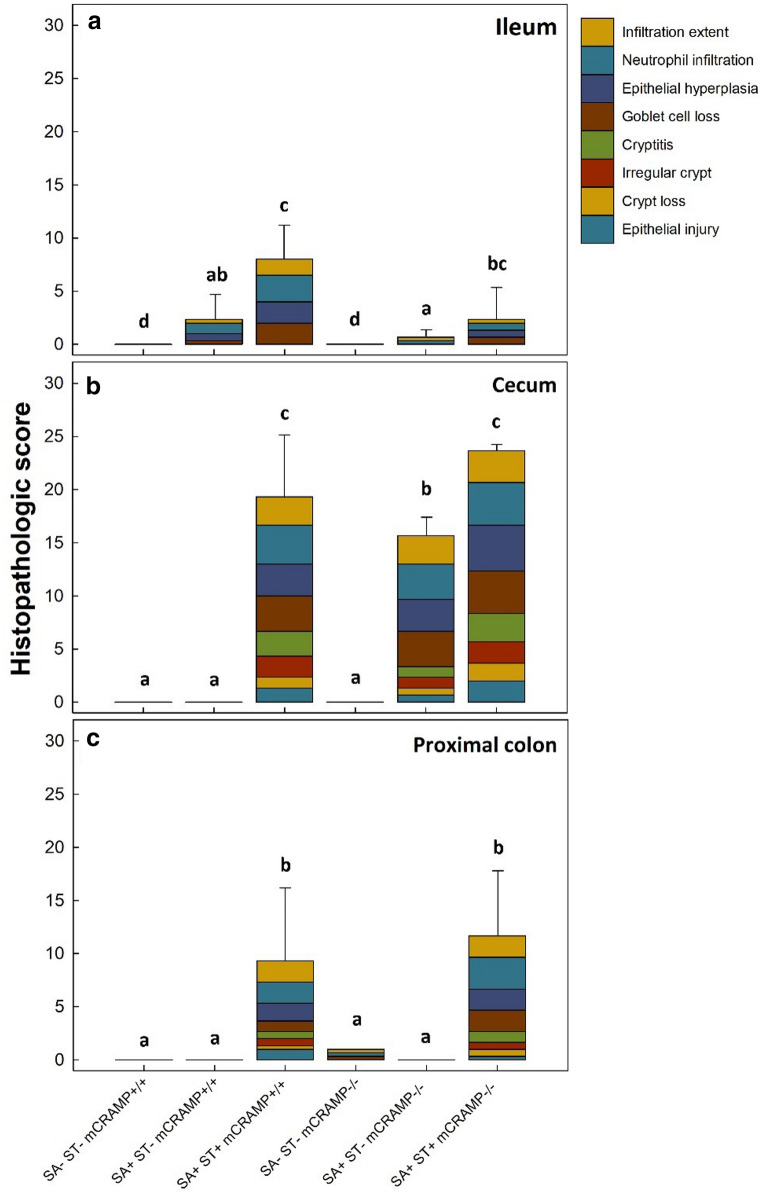
Total histopathologic scores in mCRAMP^−/−^ and mCRAMP^+/+^ mice that were inoculated with *Salmonella enterica* Typhimurium (SA+) or medium alone (SA−), and pretreated with streptomycin (ST+) or water alone (ST−) at 48 h post-inoculation. **a** Ileum; **b** Cecum; **c** Proximal Colon. Vertical lines associated with markers are standard errors of the mean. Histograms not indicated with same letter differ (P ≤ 0.050)

**Fig. 2:**
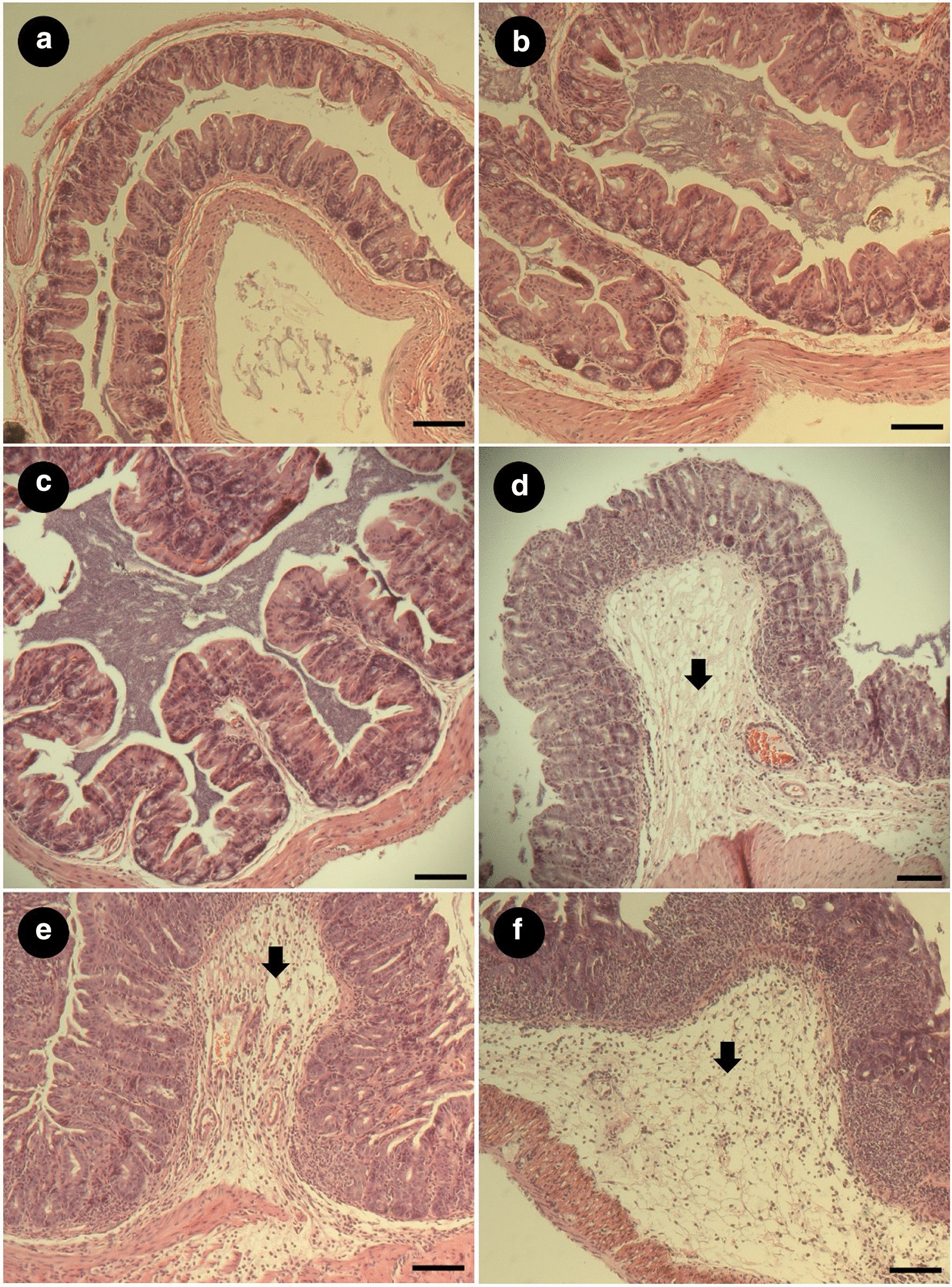
Histological representation of cecum tissue from mCRAMP^+/+^ or mCRAMP^−/−^ mice that were inoculated with *Salmonella enterica* Typhimurium (SA+) or medium alone (SA−), and pretreated with streptomycin (ST+) or water alone (ST−) at 48 h post-inoculation. Arrows indicate leukocyte infiltration. **a** SA−/ST−/mCRAMP^+/+^ mice; **b** SA−/ST−/mCRAMP^−/−^ mice; **c** SA+/ST−/mCRAMP^+/+^ mice; **d** SA+/ST−/mCRAMP^−/−^ mice; **e** SA+/ST+/mCRAMP^+/+^ mice; **f** SA+/ST+/mCRAMP^−/−^ mice. Bar = 1.0 mm

Histopathologic injury was observed in the intestines of SA+/ST−/mCRAMP^−/−^, SA+/ST+/mCRAMP^−/−^, and SA+/ST+/mCRAMP^+/+^ mice. These treatments showed higher histopathological changes (P ≤ 0.004) in the ileum, cecum, and colon at 48 h post-inoculation (hpi) than at 24 hpi (Additional file 1: Fig. S1). Although intestinal damage extended distally from the ileum to the colon, the highest level of intestinal damage was observed within the cecum (Figs. 1, 2). In the cecum, equivalent damage (P = 0.680) was observed in SA+/ST+/mCRAMP^−/−^ and SA+/ST+/mCRAMP^+/+^ mice. Although less intestinal damage was observed in SA+/ST−/mCRAMP^−/−^ than in SA+/ST+/mCRAMP^−/−^ mice (P = 0.050), these animals showed conspicuously higher (P < 0.001) histopathological scores than in SA+/ST−/mCRAMP^+/+^ mice. Thus, when mice were not pretreated with streptomycin, substantive leukocyte infiltration, hyperplasia, goblet cell loss, cryptitis, irregular crypts, crypt loss, and epithelial injury were observed only in SA+/ST−/mCRAMP^−/−^ mice (Fig. 2c, d).

### mCRAMP influenced the immune response triggered by *Salmonella* Typhimurium

**Fig. 3:**
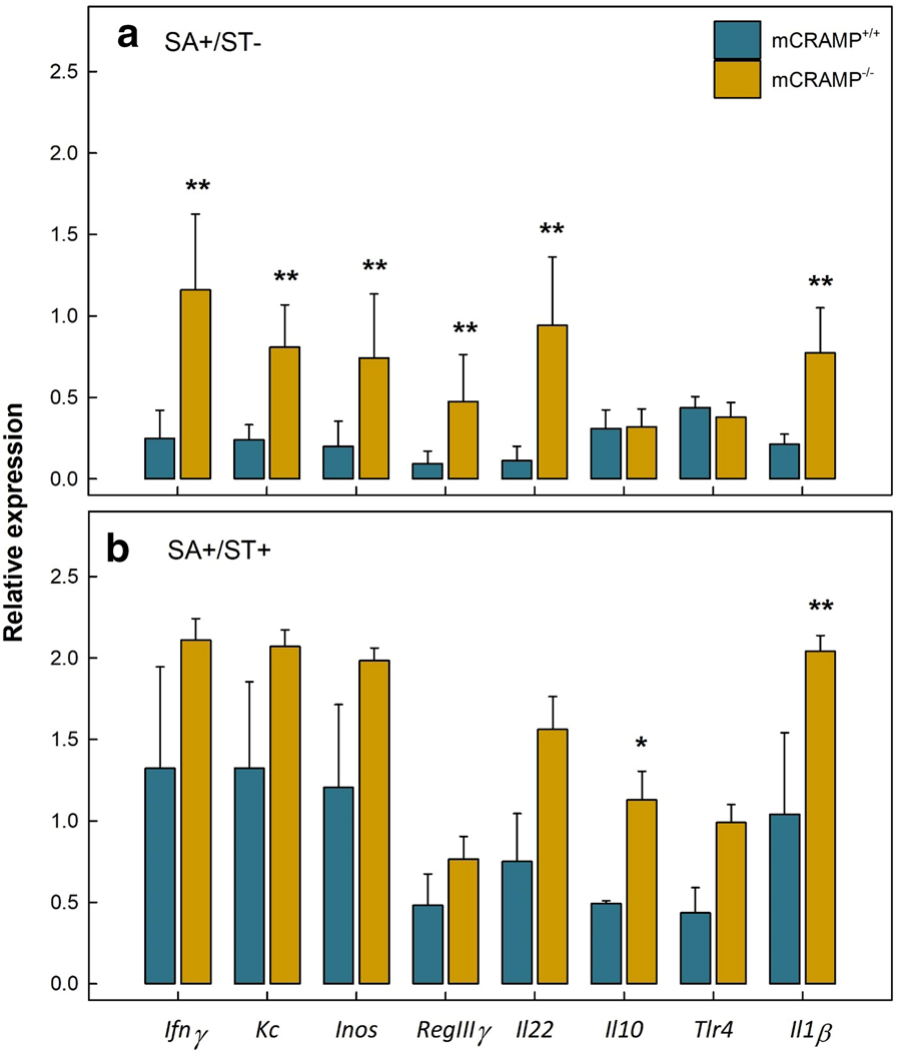
Relative gene expression in cecum of mCRAMP^+/+^ and mCRAMP^−/−^ mice that were inoculated with *Salmonella enterica* Typhimurium (SA+) at 48 h post-inoculation. **a** Mice not administered streptomycin (ST−); **b** mice pretreated with streptomycin (ST+). Vertical lines associated with markers are standard errors of the mean. Histogram bars with asterisks indicate that treatments differ (*P < 0.050, **P < 0.010)

**Fig. 4:**
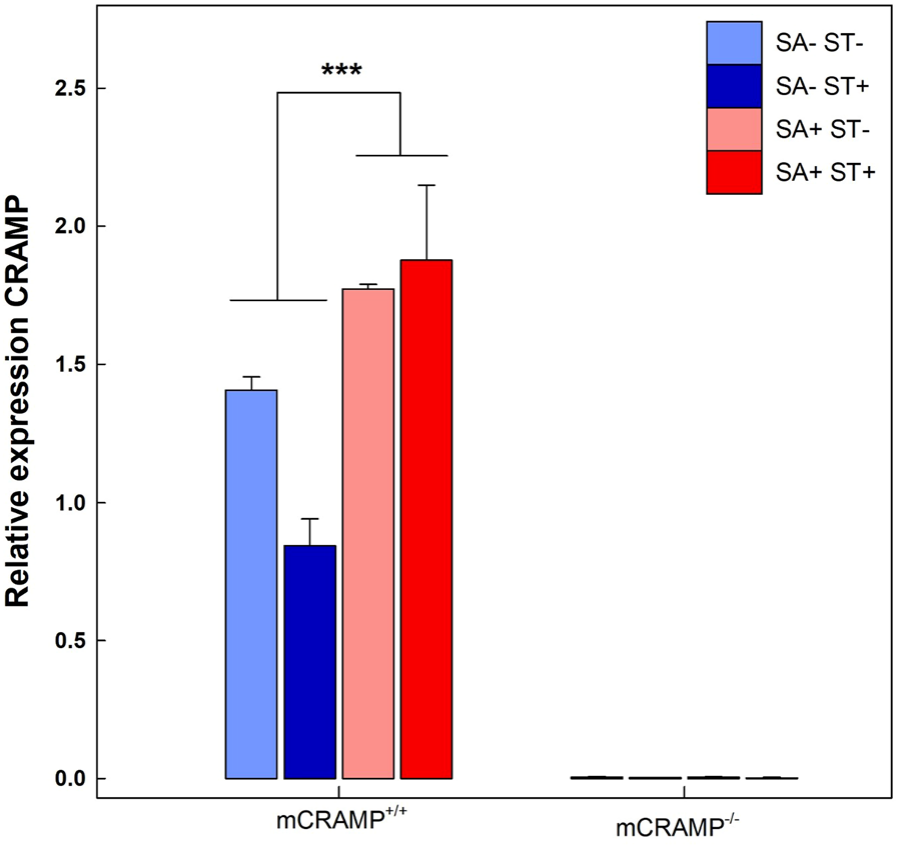
Relative expression of *mCramp* in the cecum of mCRAMP^+/+^ and mCRAMP^−/−^ mice that were inoculated with *Salmonella enterica* Typhimurium (SA+) or medium alone (SA−), and pretreated with streptomycin (ST+) or water alone (ST−) at 48 h post-inoculation. Vertical lines associated with markers are standard errors of the mean. Histogram bars with asterisks indicate that treatments differ (***P < 0.001)

Differential mRNA expression was evaluated in ileal and cecal samples of mice. In the ileum, *Kc* (P < 0.001), *Inos* (P < 0.001), *Il10* (P = 0.004), *RegIIIγ* (P < 0.001), and *Il18* (P = 0.002) were upregulated in all SA+ mice (i.e. SA+/ST−/mCRAMP^−/−^, SA+/ST+/mCRAMP^−/−^, SA+/ST−/mCRAMP^+/+^, and SA+/ST+/mCRAMP^+/+^) at 48 hpi (*data not shown*). In the cecum at 48 hpi, higher (P = 0.051) levels of gene expression were observed in both SA+/ST+/mCRAMP^−/−^ and SA+/ST+/mCRAMP^+/+^ mice relative to SA+/ST−/mCRAMP^−/−^ and SA+/ST−/mCRAMP^+/+^ mice (Fig. 3a, b). Upregulation of *Ifnγ* (P = 0.044), *Kc* (P = 0.041), *Inos* (P = 0.049), *RegIIIγ* (P = 0.024), *Il22* (P = 0.015), and *Il1β* (P = 0.045) was observed in the ceca of SA+/ST−/mCRAMP^−/−^ relative to SA+/ST−/mCRAMP^+/+^ mice at 48 hpi (Fig. 3a). *Il10* (P = 0.051) and *Il1β* (P = 0.049) were upregulated in SA+/ST+/mCRAMP^−/−^ relative to SA+/ST+/mCRAMP^+/+^ mice (Fig. 3b). A higher expression of *Ifnγ* (P ≤ 0.014), *Kc* (P ≤ 0.007), *Inos* (P ≤ 0.010), *RegIIIγ* (P ≤ 0.006), *Il22* (P ≤ 0.038)*, Il10* (P ≤ 0.001), *Tlr4* (P ≤ 0.002), and *Il1β* (P ≤ 0.018) was observed in SA+/ST+/mCRAMP^−/−^ and SA+/ST−/mCRAMP^−/−^ mice relative to SA−/ST−/mCRAMP^−/−^ (Additional file 1: Fig. S2A). Only SA+/ST+/mCRAMP^+/+^ mice showed a higher expression of *Ifnγ* (P = 0.048), *Kc* (P < 0.001), *Inos* (P < 0.001), and *Il1β* (P = 0.002) relative to SA−/ST−/mCRAMP^+/+^ animals (Additional file 1: Fig. S2B). Moreover, no difference (P ≥ 0.530) was observed in expression of immune genes among SA+/ST−/mCRAMP^+/+^, SA−/ST+/mCRAMP^+/+^, SA−/ST−/mCRAMP^+/+^, SA−/ST+/mCRAMP^−/−^, and SA−/ST−/mCRAMP^−/−^ mice. Relative expression of *mCramp* was evaluated in the ileum and cecum at 24 and 48 hpi, and no expression of the gene was observed in mCRAMP^−/−^ mice (i.e. SA−/ST−/mCRAMP^−/−^, SA−/ST+/mCRAMP^−/−^, SA+/ST−/mCRAMP^–/−^, and SA+/ST+/mCRAMP^−/−^) (Fig. 4). At 24 hpi, no differences (P ≤ 0.907) in levels of expression of *mCramp* were observed in the ileum or cecum of SA−/ST−/mCRAMP^+/+^, SA−/ST+/mCRAMP^+/+^, SA+/ST−/mCRAMP^+/+^, and SA+/ST+/mCRAMP^+/+^ mice (*data not shown*). However, higher expression (P = 0.001) of *mCramp* was observed in the cecum of SA+/ST−/mCRAMP^+/+^, and SA+/ST+/mCRAMP^+/+^ mice as compared to SA−/ST−/mCRAMP^+/+^ and SA−/ST+/mCRAMP^+/+^ mice at 48 hpi (Fig. 4). No significant differences (P ≥ 0.266) were observed among treatments in the expression of *Il4, Tgfβ, Tlr2, Tlr5, Zo1, Occludin and Muc2 (data not shown)*.

### Streptomycin and mCRAMP modified *Salmonella* Typhimurium densities within digesta and associated with mucosa in the cecum, but not in the ileum, proximal colon, or liver

**Fig. 5:**
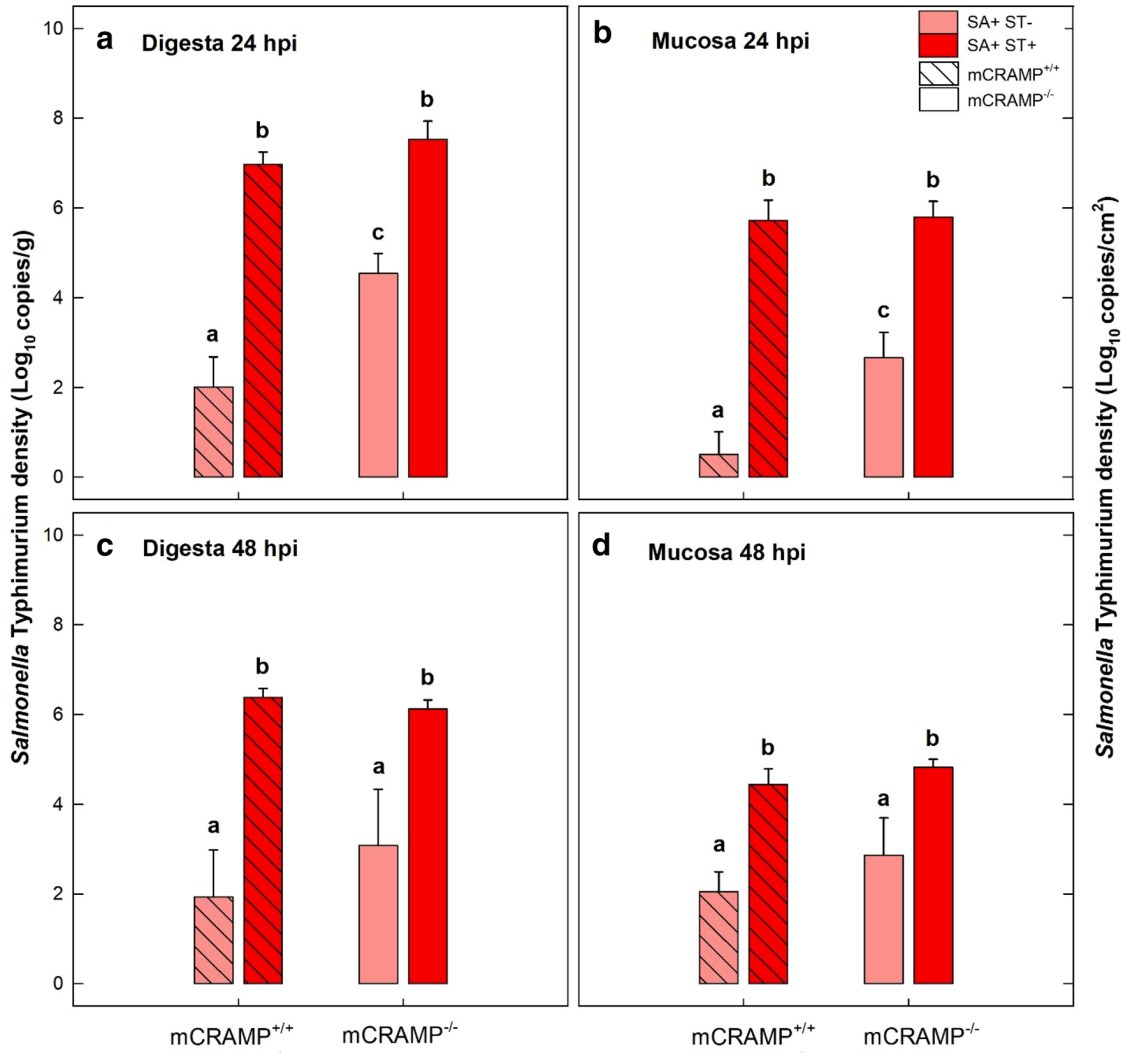
*Salmonella* densities from cecal digesta and associated with mucosa of mice 24 and 48 h post-inoculation (hpi) with *Salmonella enterica* Typhimurium (SA+), and pretreated with streptomycin (ST+) or water alone (ST−). **a** Digesta at 24 hpi; **b** mucosa-associated at 24 hpi; **c** digesta at 48 hpi; **d** mucosa-associated at 48 hpi. No *Salmonella* was detected in cecal digesta or associated with mucosa of ST− mice. Vertical lines associated with histogram bars represent standard errors of the mean. Histogram bars not indicated with the same letter differ (P < 0.050)

No *S.* Typhimurium was isolated from digesta, mucosa, or liver samples of SA− mice (i.e. SA−/ST−/mCRAMP^−/−^, SA−/ST+/mCRAMP^−/−^, SA−/ST−/mCRAMP^+/+^, and SA−/ST+/mCRAMP^+/+^). In contrast, the pathogen was isolated from all collected tissues of SA+ mice (i.e. SA+/ST−/mCRAMP^−/−^, SA+/ST+/mCRAMP^−/−^, SA+/ST−/mCRAMP^+/+^, and SA+/ST+/mCRAMP^+/+^), with the highest densities (P = 0.057) observed in digesta and associated with the mucosa of the cecum at 24 hpi. Densities of *S.* Typhimurium within digesta and associated with the mucosa in the ileum, cecum, and proximal colon tended to decrease between the 24 and 48 hpi end points (*data not shown*). In contrast, densities of the bacterium in the liver increased (P = 0.011) between the 24 and 48 hpi end points (*data not shown*). Higher densities of *S.* Typhimurium (P ≤ 0.001) were observed in SA+/ST+/mCRAMP^−/−^ and SA+/ST+/mCRAMP^+/+^ mice as compared to SA+/ST−/mCRAMP^−/−^ and SA+/ST−/mCRAMP^+/+^ mice (Fig. 5). No differences (P ≥ 0.418) were observed in *S.* Typhimurium densities between SA+/ST+/mCRAMP^+/+^ and SA+/ST+/mCRAMP^−/−^ mice at either 24 or 48 hpi. In contrast, higher (P ≤ 0.017) densities of *S.* Typhimurium were observed in SA+/ST−/mCRAMP^−/−^ as compared to SA+/ST−/mCRAMP^+/+^ mice at 24 hpi (Fig. 5a, b). There was no effect (P ≥ 0.909) of mCRAMP or antibiotic administration on densities of *S*. Typhimurium within the ileum (*data not shown*). In contrast, higher densities (P < 0.001) of *S.* Typhimurium were observed in the proximal colon of SA+/ST+/mCRAMP^−/−^ and SA+/ST+/mCRAMP^+/+^ mice (*data not shown*). No differences (P ≥ 0.381) were observed in densities of *S*. Typhimurium in the liver among SA+ mice (i.e. SA+/ST−/mCRAMP^−/−^, SA+/ST+/mCRAMP^−/−^, SA+/ST−/mCRAMP^+/+^, and SA+/ST+/mCRAMP^+/+^) (*data not shown*).

### *Salmonella* Typhimurium infection modified immune proteins in the small intestine and serum

Immune protein concentrations were measured in serum as well as in the ileal tissues. In the ileum, higher concentrations (P ≤ 0.004) of myeloperoxidase (MPO) and keratinocyte-derived chemokine (KC) were observed in SA+ mice (i.e. SA+/ST−/mCRAMP^−/−^, SA+/ST+/mCRAMP^−/−^, SA+/ST−/mCRAMP^+/+^, and SA+/ST+/mCRAMP^+/+^) (Additional file 1: Fig. S3), and concentrations increased (P ≤ 0.025) at 48 hpi. No differences (P ≥ 0.729) in the concentrations of MPO or KC were observed between SA+/ST+/mCRAMP^−/−^ and SA+/ST−/mCRAMP^−/−^ mice. In contrast, a higher (P = 0.020) concentration of KC was observed in the ileum of SA+/ST−/mCRAMP^−/−^ mice as compared to SA+/ST−/mCRAMP^+/+^ mice at 48 hpi. In the serum, higher (P ≤ 0.001) concentrations of MPO were observed in SA+ mice (i.e. SA+/ST−/mCRAMP^−/−^, SA+/ST+/mCRAMP^−/−^, SA+/ST−/mCRAMP^+/+^, and SA+/ST+/mCRAMP^+/+^), and concentrations were higher (P ≤ 0.007) at 48 hpi (Additional file 1: Fig S4). mCRAMP was not detected in the serum or ileum of mCRAMP^−/−^ mice (i.e. SA−/ST−/mCRAMP^−/−^, SA−/ST+/mCRAMP^−/−^, SA+/ST−/mCRAMP^−/−^, and SA+/ST+/mCRAMP^−/−^). No differences (P ≥ 0.991) were observed in mCRAMP concentration in serum or ileum among mCRAMP^+/+^ mice (i.e. SA−/ST−/mCRAMP^+/+^, SA−/ST+/mCRAMP^+/+^, SA+/ST−/mCRAMP^+/+^, and SA+/ST+/mCRAMP^+/+^) (*data not shown*).

### Splenic immune cell populations varied over time

Immune cell populations within the spleen were comparatively analyzed for proportions of T cell subsets, NK cells, and differentiated monocytes and neutrophils. The proportion of splenic CD45^+^ leukocytes expressing CD18 in mCRAMP^−/−^ mice at 24 hpi showed no difference (P ≥ 0.482) among treatments (i.e. SA−/ST−/mCRAMP^−/−^, SA−/ST+/mCRAMP^−/−^, SA+/ST−/mCRAMP^−/−^, and SA+/ST+/mCRAMP^−/−^) (Additional file 1: Fig. S5C). In contrast, at 24 hpi, the population of CD45^+^ leukocytes expressing CD18 was reduced (P ≤ 0.035) in SA+/ST+/mCRAMP^+/+^ and SA−/ST+/mCRAMP^+/+^ mice (Additional file 1: Fig. S5C). At 48 hpi, this same population was conspicuously reduced (P ≤ 0.014) in SA+ mice (i.e. SA+/ST−/mCRAMP^−/−^, SA+/ST+/mCRAMP^−/−^, SA+/ST−/mCRAMP^+/+^, and SA+/ST+/mCRAMP^+/+^) as compared to SA− mice (i.e. SA−/ST−/mCRAMP^−/−^, SA−/ST+/mCRAMP^−/−^, SA−/ST−/mCRAMP^+/+^, and SA−/ST+/mCRAMP^+/+^) (Additional file 1: Fig. S5D). The percentage of CD18^+^CD11b^+^ leukocytes increased (P ≤ 0.001) in SA+ mice (i.e. SA+/ST−/mCRAMP^−/−^, SA+/ST+/mCRAMP^−/−^, SA+/ST−/mCRAMP^+/+^, and SA+/ST+/mCRAMP^+/+^) relative to SA− mice (i.e. SA−/ST−/mCRAMP^−/−^, SA−/ST+/mCRAMP^−/−^, SA−/ST−/mCRAMP^+/+^, and SA−/ST+/mCRAMP^+/+^) at 24 and 48 hpi (Additional file 1: Fig. S5A-B). The proportion of these leukocytes was reduced (P ≤ 0.023) at 48 hpi relative to 24 hpi independent of treatment (Additional file 1: Fig. S5A-B). The percentage of CD18^+^CD11b^+^ Ly-6C^+^Ly-6G^+^ neutrophils increased (P ≤ 0.012) in SA+ mice (i.e. SA+/ST−/mCRAMP^−/−^, SA+/ST+/mCRAMP^−/−^, SA+/ST−/mCRAMP^+/+^, and SA+/ST+/mCRAMP^+/+^) relative to SA− mice (i.e. SA−/ST−/mCRAMP^−/−^, SA−/ST+/mCRAMP^−/−^, SA−/ST−/mCRAMP^+/+^, and SA−/ST+/mCRAMP^+/+^) (Additional file 1: Fig. S5E-F). No differences (P ≥ 0.164) were observed in other immune cell populations (Additional file 1: Table S1).

### The liver metabolite profile was modified by *Salmonella* Typhimurium infection

**Fig. 6:**
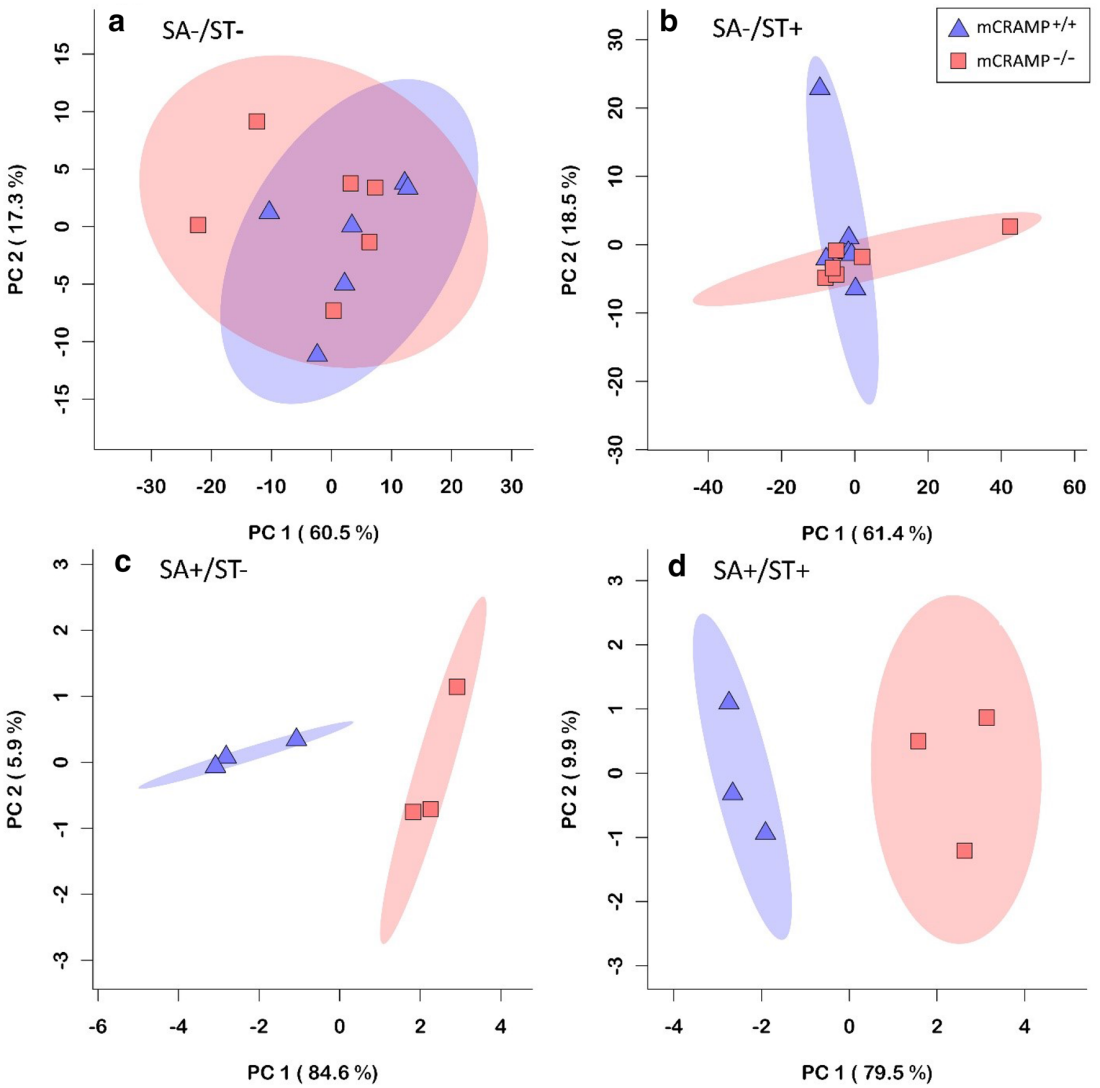
Metabolite profiles of mice livers. Principal component analysis plots showing separation between mCRAMP^−/−^ and mCRAMP^+/+^ mice, inoculated with *Salmonella* Typhimurium (SA+) or medium alone (SA−), and pretreated with streptomycin (ST+) or water (ST−). **a** SA−/ST−/mCRAMP^−/−^ vs SA−/ST−/mCRAMP^+/+^ mice at 24 and 48 h post-inoculation (hpi); **b** SA−/ST+/mCRAMP^−/−^ vs SA−/ST+/mCRAMP^+/+^ mice at 24 hpi and 48 hpi; **c** SA+/ST−/mCRAMP^−/−^ vs SA+/ST−/mCRAMP^+/+^ mice at 24 hpi; **d** SA+/ST+/mCRAMP^−/−^ vs SA+/ST+/mCRAMP^+/+^ mice at 24 hpi

**Fig. 7:**
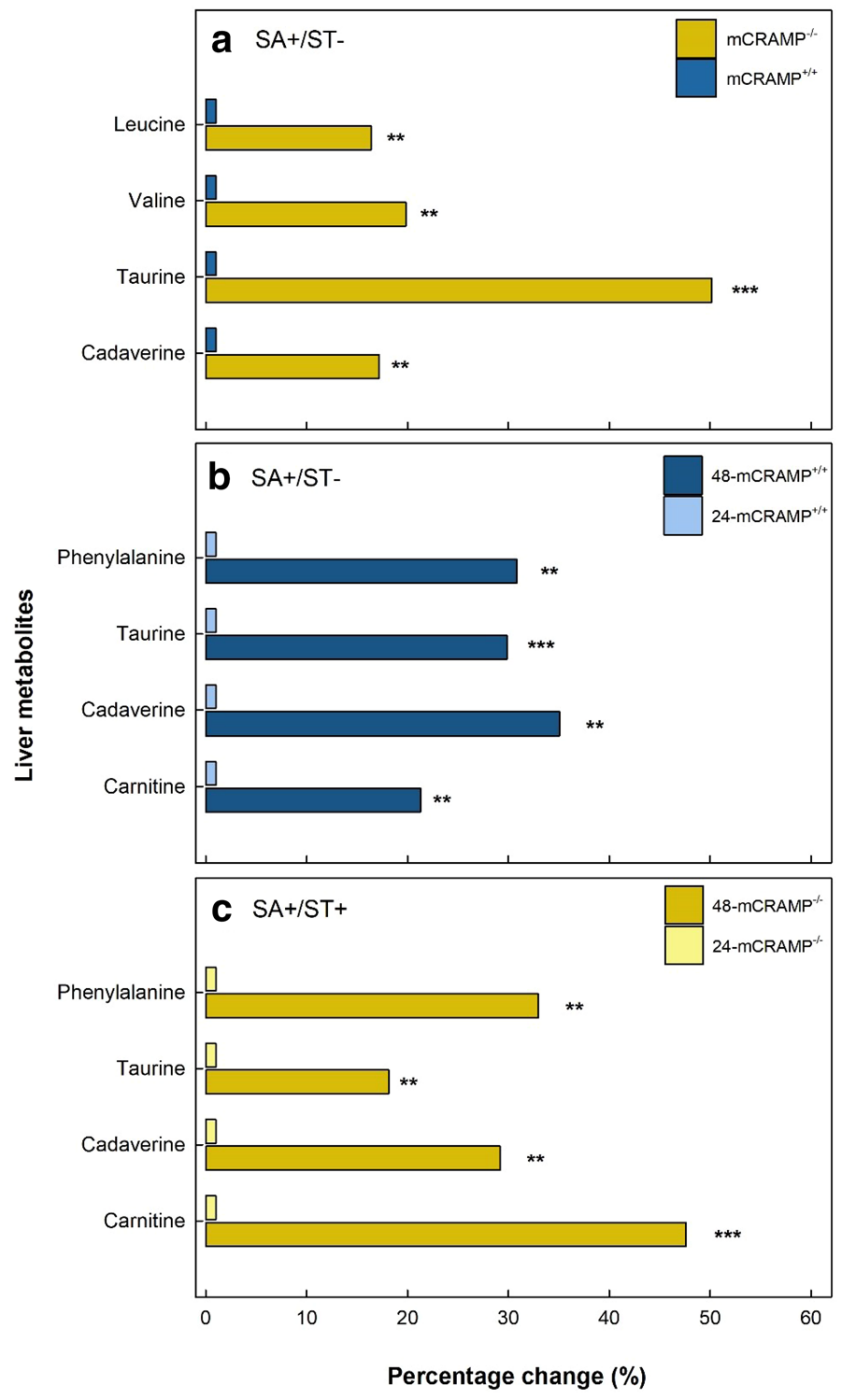
Percentage change of discriminated liver metabolites in mCRAMP^−/−^ and mCRAMP^+/+^ mice that were inoculated with *Salmonella* Typhimurium (SA+), and pretreated with streptomycin (ST+) or water alone (ST−). **a** SA+/ST−/mCRAMP^−/−^ vs SA+/ST−/mCRAMP^+/+^ mice at 24 h post-inoculation (hpi). **b** SA+/ST−/mCRAMP^+/+^ mice at 24 and 48 hpi. **c** SA+/ST+/mCRAMP^−/−^ mice at 24 and 48 hpi. Histogram bars with asterisks indicate that treatments differ (*P < 0.050, **P < 0.010)

Water-soluble metabolites were extracted from the right medial lobe of the liver and analyzed by H-Nuclear Magnetic Resonance (NMR) spectroscopy to evaluate changes in the metabolome associated with streptomycin administration, *Salmonella* infection, and absence of mCRAMP. A comparison between 24 hpi and 48 hpi showed no differences among SA−/ST−/mCRAMP^−/−^, SA−/ST−/mCRAMP^+/+^, SA−/ST+/mCRAMP^−/−^, and SA−/ST+/mCRAMP^+/+^ mice (*data not shown*); therefore, the 24 and 48 hpi treatments were grouped. There was no separation in metabolite profiles between SA−/ST−/mCRAMP^−/−^ and SA−/ST−/mCRAMP^+/+^ mice (Fig. 6a). There was also no separation in profiles between SA−/ST+/mCRAMP^−/−^ and SA−/ST+/mCRAMP^+/+^ mice (Fig. 6b). In contrast, SA+/ST−/mCRAMP^−/−^ and SA+/ST−/mCRAMP^+/+^ mice showed separation in metabolite profiles at 24 hpi (Fig. 6c), but not at 48 hpi. In this regard, increases of cadaverine (P = 0.022), taurine (P = 0.047), valine (P = 0.037), and leucine (P = 0.038) were observed in SA+/ST−/mCRAMP^−/−^ at 24 hpi (Fig. 7a). A temporal comparison of metabolite profiles in SA+/ST−/mCRAMP^+/+^ mice showed increases of phenylalanine (P = 0.028), taurine (P = 0.003), cadaverine (P = 0.014), and carnitine (P = 0.030) at 48 hpi relative to 24 hpi (Fig. 7b). Metabolic profiles of SA+/ST+/mCRAMP^+/+^ and SA+/ST+/mCRAMP^−/−^ mice showed separation at 24 hpi (Fig. 6d). Evaluation of specific metabolites showed increases of taurine (P ≤ 0.033) and carnitine (P ≤ 0.010) in SA+/ST+/mCRAMP^−/−^ mice at 24 and 48 hpi as compared to SA+/ST+/mCRAMP^+/+^ mice (*data not shown*). Evaluation of metabolite profiles over time in SA+/ST+/mCRAMP^−/−^ mice showed more alterations at 48 hpi, including increases in phenylalanine (P = 0.026), taurine (P = 0.013), cadaverine (P = 0.028), and carnitine (P = 0.004) (Fig. 7c).

### The composition of the bacterial community, but not diversity in cecal digesta, was subtly different in mCRAMP-knockout mice not inoculated with *Salmonella* Typhimurium or administered streptomycin sulfate

Characterization of bacterial communities in the cecum digesta was carried out by next generation sequencing (NGS) using an Illumina MiSeq platform. The composition of the microbiota differed subtly between SA−/ST−/mCRAMP^+/+^ and SA−/ST−/mCRAMP^−/−^ mice. The microbiota was dominated by *Firmicutes*, representing 51.3% and 69.2% of the community for SA−/ST−/mCRAMP^+/+^ and SA−/ST−/mCRAMP^−/−^ mice, respectively (Additional file 1: Fig. S6A). A higher relative abundance of *Bacteroidetes* was observed in SA−/ST−/mCRAMP^+/+^ mice (34.3%) as compared to SA−/ST−/mCRAMP^−/−^ mice (7.1%) (Additional file 1: Fig. S6A). At the family level of resolution, these differences were reflected by a higher relative abundance of *Bacteroideaceae* in SA−/ST−/mCRAMP^+/+^ mice and a higher abundance of *Muribaculaceae* in SA−/ST−/mCRAMP^−/−^ mice (Additional file 1: Fig. S6B).

**Fig. 8:**
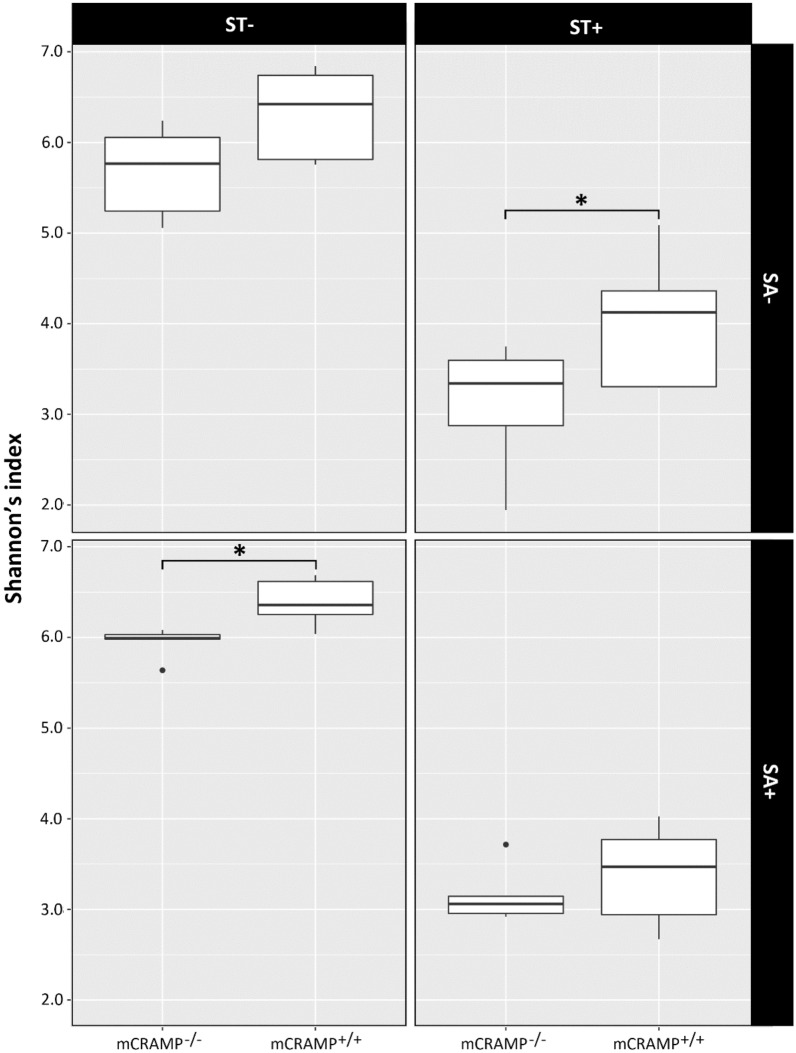
Alpha-diversity of bacterial communities in digesta from cecum of mCRAMP^−/−^ and mCRAMP^+/+^ mice that were inoculated with *Salmonella enterica* Typhimurium (SA+) or medium alone (SA−), and pretreated with streptomycin (ST+) or water alone (ST−). Samples were obtained from mice at 24 and 48 h post-inoculation. The boxes represent interquartile ranges with the black line indicating the median value. The size of the boxes denote the distribution within a confidence of 95%, vertical lines represent the total distribution of the data. Boxes with an asterisk indicate that treatments differ (*P < 0.050)

**Fig. 9:**
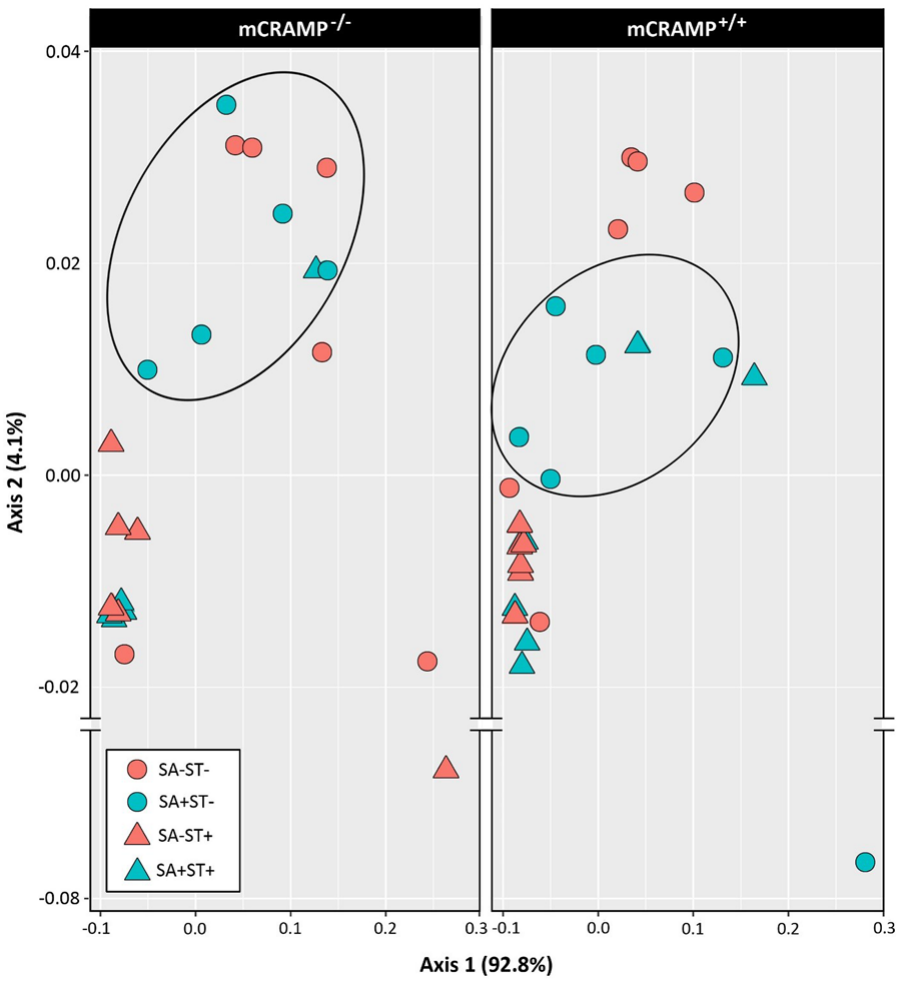
Principal component analysis showing unweighted UniFrac distances of bacterial communities in cecal digesta of mCRAMP^−/−^ and mCRAMP^+/+^ mice that were inoculated with *Salmonella enterica* Typhimurium (SA+) or medium alone (SA−), and pretreated with streptomycin (ST+) or water alone (ST−). Samples were obtained from mice at 24 and 48 h post-inoculation. Ellipsoids highlight clustering of treatments

There were no differences in Shannon’s index of alpha diversity (Fig. 8), Pielou’s evenness, number of amplicon sequence variants (ASVs) or Faith phylogenetic diversity between SA−/ST−/mCRAMP^−/−^ and SA−/ST−/mCRAMP^+/+^ mice at either 24 hpi (P ≥ 0.248) or 48 hpi (P ≥ 0.126) (Additional file 1: Table S2). Additionally, there were no differences in beta diversity as evaluated by unweighted and weighted principal component analysis between SA−/ST−/mCRAMP^−/−^ and SA−/ST−/mCRAMP^+/+^ mice at 24 hpi (P ≥ 0.201) or 48 hpi (P ≥ 0.175) (Fig. 9) (Additional file 1: Table S2).

### Administration of streptomycin sulfate modified the composition and diversity of the cecal digesta microbiota in mCRAMP^−/−^ and mCRAMP^+/+^ mice

The administration of streptomycin sulfate conspicuously affected both the diversity and composition of the enteric microbiota. ASV counts (P ≤ 0.006), Shannon’s index (P < 0.003) (Fig. 8), Pielou’s evenness (P < 0.003), and Faith phylogenetic diversity (P ≤ 0.010) were reduced in ST+ mice (i.e. SA−/ST+/mCRAMP^−/−^, SA+/ST+/mCRAMP^−/−^, SA−/ST+/mCRAMP^+/+^, and SA+/ST+/mCRAMP^+/+^) as compared to ST− mice (i.e. SA−/ST−/mCRAMP^−/−^, SA+/ST−/mCRAMP^−/−^, SA−/ST−/mCRAMP^+/+^, and SA+/ST−/mCRAMP^+/+^) (Additional file 1: Table S2). Additionally, weighted (P ≤ 0.009) and unweighted (P ≤ 0.008) (Fig. 9) principal coordinate analysis showed differences between ST+ mice (i.e. SA−/ST+/mCRAMP^−/−^, SA+/ST+/mCRAMP^−/−^, SA−/ST+/mCRAMP^+/+^, and SA+/ST+/mCRAMP^+/+^) with ST− mice (i.e. SA−/ST−/mCRAMP^−/−^, SA+/ST−/mCRAMP^−/−^, SA−/ST−/mCRAMP^+/+^, and SA+/ST−/mCRAMP^+/+^) (Additional file 1: Table S2). At a phyla level of resolution, SA−/ST+/mCRAMP^+/+^ mice showed a decrease in the relative abundance of *Bacteroidetes* and *Firmicutes* with an increase in the relative abundance of *Proteobacteria* and *Verrucomicrobia* (Additional file 1: Fig. S6A). In contrast, SA−/ST+/mCRAMP^−/−^ mice exhibited a higher abundance of *Firmicutes* and *Verrucomicrobia* phyla (Additional file 1: Fig. S6A). SA+/ST+/mCRAMP^−/−^ mice showed a conspicuous shift in the composition of the microbiota, with a higher abundance of *Proteobacteria* (97.5%) as compared to SA+/ST+/mCRAMP^+/+^ mice (65%) (Additional file 1: Fig. S6A). All of the bacterial ASVs identified as *Proteobacteria* in infected mice were members of the *Enterobacteriaceae* (Additional file 1: Fig. S6B). No differences (P ≥ 0.295) were observed in alpha or beta diversity between SA+/ST+/mCRAMP^+/+^ and SA+/ST+/mCRAMP^−/−^ mice (Additional file 1: Table S2).

### Bacterial communities differed between mCRAMP^−/−^ and mCRAMP^+/+^ mice infected with *Salmonella* Typhimurium and not administered streptomycin

Gneiss analysis revealed that bacterial communities in the ceca of SA+/ST−/mCRAMP^−/−^ and SA+/ST−/mCRAMP^+/+^ mice differed. In this regard, SA+/ST−/mCRAMP^−/−^ mice had 32 taxa that differed in cecal abundance in comparison to SA+/ST−/mCRAMP^+/+^ mice. Of these taxa, *Akkermansia* and *Anaeroplasma* were conspicuously more abundant in SA+/ST−/mCRAMP^−/−^ mice (Additional file 1: Fig. S6B). Differences in alpha diversity corresponded to bacterial composition; SA+/ST−/mCRAMP^+/+^ mice exhibited a higher Shannon diversity index (P = 0.017) (Fig. 8) and Faith’s phylogeny diversity (P = 0.017) than SA+/ST−/mCRAMP^−/−^ mice. In addition, a difference in beta diversity was observed between SA+/ST−/mCRAMP^+/+^ and SA+/ST−/mCRAMP^−/−^ mice for both unweighted UniFrac principal coordinate analysis (P = 0.003) (Fig. 9), as well as weighted UniFrac principal coordinate analysis (P = 0.003).

## Discussion

The protective role of host-defense peptides has been widely studied [14, 15, 39]. Murine cathelicidin-related antimicrobial peptide (mCRAMP) has been shown to impair infection by invasive pathogens within the skin [13], urinary tract [14], and gastrointestinal tract [3]. In vitro studies have shown an antimicrobial effect of mCRAMP directed to *S.* Typhimurium [10], and additionally, the intracellular expression of this cathelicidin in macrophages has been shown to impair *S.* Typhimurium replication [40]. However, no studies to our knowledge have been conducted in vivo to evaluate the interplay among cathelicidin, the host, the microbiota, and *S.* Typhimurium. Since secretion of mCRAMP has been reported to modulate the composition of the enteric microbiota [21], understanding the role that cathelicidin has on the host–pathogen-microbiota interaction is required to evaluate its role in colonization resistance. In the current study, we inoculated mice with the highly virulent *S.* Typhimurium DT104 strain [41] or buffer alone (i.e. uninfected control treatment). To ascertain the role that mCRAMP has on salmonellosis, we temporally evaluated immune responses, the structure of the enteric microbiota, and the metabolome of the liver in mCRAMP^−/−^ and mCRAMP^+/+^ mice ± oral administration of streptomycin sulfate.

### mCRAMP deficient mice exhibited alterations to the intestinal wall structure following infection

Treatment of mice with streptomycin sulfate to create a dysbiosis is required to generate salmonellosis that is characteristically similar to enteric inflammation observed in human beings [42]. Importantly, we observed that SA+/ST+ mice developed enteric salmonellosis. Intestinal damage was more prominent in the large intestine, especially in the cecum, which is consistent with previous studies [43]. At 48 hpi, the architecture of the cecal mucosa was conspicuously disrupted in SA+/ST+/mCRAMP^−/−^ and SA+/ST+/mCRAMP^+/+^ mice, which included epithelial hyperplasia, reduced numbers of goblet cells, epithelial injury, and marked polymorphonuclear infiltration of the submucosa, lamina propria, and the epithelium. These histopathologic changes to cecal tissues have previously been described in dysbiotic mice infected with the bacterium [33]. Significantly, we observed similar degree of intestinal damage in SA+/ST−/mCRAMP^−/−^ relative to SA+/ST+/mCRAMP^+/+^ mice demonstrating that mCRAMP^−/−^ did not depend on the pretreatment with streptomycin to develop intestinal salmonellosis, which suggested that mCRAMP-deficient mice were more susceptible to the bacterium.

### mCRAMP deficiency modifies the host immune response

Cathelicidins have been shown to participate in host immune responses by inducing chemoattraction of granulocytes or by enhancing the adaptive immune system [17]. Therefore, evaluation of immune marker modulation is essential to understand *S.* Typhimurium infection and the role of cathelicidin in this enteric disease. Although the immune responses triggered after infection by *S.* Typhimurium in mice have been previously described [38, 44], to the best of our knowledge no studies have yet been conducted to describe the role of mCRAMP on immune responses developed locally (intestine) and systemically (spleen and liver) due to infection by *S*. Typhimurium. The greatest histopathologic changes were observed in the cecum, and we thus evaluated mRNA expression in the cecum as well as in the ileum. Upregulation of immune markers was observed in the cecum of SA+/ST+/mCRAMP^+/+^, SA+/ST−/mCRAMP^−/−^, and SA+/ST+/mCRAMP^−/−^ mice at 48 hpi. More specifically, we observed upregulation of the pattern recognition receptor *Tlr4*, which plays an essential role in recognition of *Salmonella* lipopolysaccharide and the consequent elimination of the pathogen [45]. At 48 hpi, pro-inflammatory cytokines were highly upregulated in the cecal mucosa of SA+ relative to SA− mice. Higher expression of *Il1β, Ifnγ, Kc,* and *Il22* were identified in both SA+/ST+/mCRAMP^+/+^ and SA+/ST+/mCRAMP^−/−^ mice. Additionally, SA+/ST−/mCRAMP^−/−^ and SA+/ST+/mCRAMP^−/−^ mice showed higher expression of these cytokines relative to SA+/ST−/mCRAMP^+/+^ and SA+/ST+/mCRAMP^+/+^ mice. The higher expression of these cytokines in SA+/ST−/mCRAMP^−/−^ and SA+/ST+/mCRAMP^−/−^ mice correlates with the severe damage observed in the cecum, further supporting an increased susceptibility of mCRAMP-deficient mice to salmonellosis. Mice deficient in IL-1β succumb more easily to *Salmonella* infection, highlighting the importance of this cytokine in the mucosal immune response against this pathogen [46]. Upregulation of *Ifnγ* and *Il22* have been previously reported in *S.* Typhimurium infected mice that were pretreated with streptomycin, illustrating the pivotal role they play in the amplification of the intestinal immune response [47]. This amplification is mainly associated with neutrophil chemoattraction to the site of infection [48] following the release of keratinocyte-derived chemokine (KC) [49], and secretion of nitric oxide synthase (INOS) [47]. In the current study, we observed upregulation of both *Kc* and *Inos* in all SA+ relative to SA− mice. However, higher levels of both cytokines were observed in SA+/ST−/mCRAMP^−/−^ and SA+/ST+/mCRAMP^−/−^ mice as compared to SA+/ST−/mCRAMP^+/+^ and SA+/ST+/mCRAMP^+/+^ mice. Elevated presence of neutrophils in the submucosa, lamina propria, and epithelium were observed in SA+/ST−/mCRAMP^−/−^, SA+/ST+/mCRAMP^−/−^, and SA+/ST+/mCRAMP^+/+^ mice, which corresponds with the higher expression of the chemoattractant *Kc* observed. No differences between SA+ and SA− mice were observed in *Il18* expression in the current study. Although this cytokine can have an essential role in the host resistance to the serotype Typhimurium, this function is mainly associated to the systemic phase of the disease, particularly in splenic and liver tissues [46].

Higher expression of *mCramp* was observed in the cecum of SA+/ST−/mCRAMP^+/+^ and SA+/ST+/mCRAMP^+/+^ mice at 48 hpi. Local expression of *mCramp* has been described to be limited to the colonic epithelium [10]. However, we were able to demonstrate the presence of this antimicrobial peptide in the cecal and ileal mucosa, with the former being the location where salmonellosis was primarily manifested. The secretion of mCRAMP by neutrophils has been previously reported [10]. Therefore, the high expression that we observed at 48 hpi in SA+/ST+/mCRAMP^+/+^ mice is likely associated with the elevated cecal infiltration of neutrophils at this time point. Additionally, we observed upregulation of *RegIIIγ* in the cecum of all SA+ mice relative to SA− mice, with higher expression in SA+/ST−/mCRAMP^−/−^ and SA+/ST+/mCRAMP^−/−^mice as compared to SA+/ST−/mCRAMP^+/+^ and SA+/ST+/mCRAMP^+/+^ mice. Higher expression of this host defense peptide in response to *Salmonella* infection has been previously described [50]. In addition to pro-inflammatory responses, we also conducted evaluation of anti-inflammatory cytokines. We observed that SA+ mice had a higher expression of *Il10* in the cecal mucosa at 48 hpi. The upregulation of this cytokine was likely directed to control the exacerbated inflammatory response triggered by the pathogen [51].

Proteins involved in immune function were examined in the ileum and in serum, which showed that keratinocyte-derived chemokine (KC) and myeloperoxidase (MPO) were highly concentrated in ileum of SA+ mice at 24 hpi, and increased over time. Similar results were observed for MPO in the serum of SA+ mice. The higher concentration of MPO observed in SA+ mice can be directly associated with *S.* Typhimurium infection, since this enzyme produces hypochlorous acid (HOCl), a key cytotoxic antimicrobial product released by neutrophils against the pathogen [52]. Additionally, the higher concentration of both KC and MPO observed in the intestine at 48 hpi likely correlates to the higher infiltration of neutrophils observed at this time point. Elevated serum levels of MPO could also be associated with the higher load of *Salmonella* travelling through the blood at this time, which is consistent with previous studies [33].

In ST− mice, *Salmonella* Typhimurium colonizes Peyer’s patches and mesenteric lymph nodes, and then quickly spreads to the spleen and liver at which point the infection is considered systemic [53, 54]. A previous study showed that mice administered streptomycin prior to *S.* Typhimurium inoculation presented extensive infiltration of CD18^+^ cells into the lamina propria, and this flux of cells exhibiting a phagocytic/antigen-presenting cell phenotype has been suggested as a mechanism by which the pathogen breaches the intestinal barrier to incite a systemic response [33]. In the current study, the overall splenic population of CD18^+^ leukocytes decreased in SA+/ST+/mCRAMP^−/−^ and SA+/ST+/mCRAMP^+/+^ mice at 48 hpi, suggesting this population migrated from the spleen in both genotypes. The CD18^+^CD11b^+^ phenotype represents inflammatory cells capable of phagocytosis and antigen presentation, and this combination of cell surface receptors is required for the process of extravasation [55, 56]. The CD18^+^CD11b^+^ cell phenotype was higher in SA+ mice, and splenic populations decreased at 48 hpi, suggesting migration of CD18^+^CD11b^+^ cells from the spleen. Overall, SA+ mice maintained higher proportions of neutrophils than SA− mice, further demonstrating the impact of the infection on immune cell populations in the spleen. After 48 hpi, minimal differences were observed between the splenic immune cell populations of SA+ mice, suggesting the pretreatment with streptomycin and infection by *S*. Typhimurium had greater influences on splenic cell population structure than the presence of functional cathelicidin.

### *Salmonella* Typhymurium densities corresponded to disease progression

*Salmonella* Typhimurium densities directly correspond to histopathological changes and to the modification of the immune responses that were observed in the current study. Higher densities of the pathogen were observed at 24 hpi in the digesta and cecum mucosa of SA+/ST−/mCRAMP^−/−^, SA+/ST+/mCRAMP^−/−^, and SA+/ST+/mCRAMP^+/+^ mice, and decreased over time. Disruption of the mucosal layer is associated with higher internalization of the bacterium into the blood stream [33, 57]. Therefore, the lower densities that we observed in the digesta and mucosa at 48 hpi could be directly associated with systemic dissemination [33]. This is also in line with the increase in pathogen densities that we observed in the liver of SA+ mice at 48 hpi. Additionally, the lower bacterial densities observed at 48 hpi in the mucosa of SA+ mice could be the consequence of the immune response already established against the pathogen [44]. The absence of a difference in *Salmonella* densities that we observed between SA+/ST+/mCRAMP^−/−^ and SA+/ST+/mCRAMP^+/+^ mice could be strictly associated to the effect that streptomycin has on the microbiota [33]. The elimination of a bacterial community that can compete with the pathogen is reflected as a higher density of *Salmonella* associated with the mucosa of these animals (i.e. in SA+/ST+/mCRAMP^−/−^ and SA+/ST+/mCRAMP^+/+^ mice, in which the microbiota was equally disrupted by streptomycin administration). In contrast, when pretreatment of streptomycin was not performed, and therefore, the structure of the microbiota was conserved, mCRAMP^−/−^ mice exhibited a much higher degree of mucosal colonization by *Salmonella* in comparison to mCRAMP^+/+^ mice. This is consistent with the increased susceptibility to *Salmonella* enterocolitis that was observed in mCRAMP deficient mice.

### mCRAMP deficient mice showed more alterations in the liver metabolome following infection

We used metabolomics to characterize the effects of *S.* Typhimurium infection and streptomycin administration on the liver due to systemic infection, and importantly, to determine the role that mCRAMP plays in these changes. Results showed that the liver metabolome of SA− mice did not differ. In contrast, the liver metabolome differed significantly in SA+ mice. When the microbiota is not altered by a broad spectrum antibiotic, naïve mice inoculated with *S.* Typhimurium develop a typhoid-like disease [58], characterized by parenchymal necrosis of the liver and spleen [59]. To address if SA+/ST−/mCRAMP^−/−^ and SA+/ST−/mCRAMP^+/+^ mice differed in the development of typhoid-like fever, we compared their liver metabolic profiles. We observed that the liver metabolome of SA+/ST−/mCRAMP^−/−^ mice was conspicuously more affected than SA+/ST−/mCRAMP^+/+^ mice at 24 hpi, but the difference between the treatments abated by 48 hpi. SA+/ST+/mCRAMP^−/−^ mice also showed a higher degree of change in the liver metabolome relative to SA+/ST+/mCRAMP^+/+^ at 24 and 48 hpi. Moreover, an increase in valine, leucine, taurine, and cadaverine was observed in the livers of SA+/ST−/mCRAMP^−/−^ relative to SA+/ST−/mCRAMP^+/+^ mice at 24 hpi. While lower plasma levels of branched-chain amino-acids (BCCA) (e.g. valine, leucine) have previously been reported in mice with septicemia, such as typhoid fever [60, 61], the same amino acids tend to increase in liver under infection [62]. Since BCCA have been observed to induce an immune response enhancing neutrophil function [63], their higher concentration in SA+/ST−/mCRAMP^−/−^ mice could be due to an enhanced immune response as a result of the higher degree of colonization by the pathogen. An elevated concentration of taurine in the intestine is associated with higher levels of oxidants, and this may aid in preventing tissue injury under conditions of inflammation [64–66]. A study conducted in intestinal epithelial cells showed a correlation between the production of the polyamine cadaverine and inhibition of polymorphonuclear transmigration induced by *Shigella* infection via avoidance of the immune response reaction triggered by epithelial cells [67]. Therefore, the higher concentration of taurine and cadaverine observed in SA+/ST−/mCRAMP^−/−^ mice is likely the result of an exacerbated immune response triggered against the pathogen. *Salmonella* dissemination and liver damage triggered by the bacterium progress over time [57], and the significant time effect that we observed in the liver metabolome is consistent with this conclusion. More specifically, we observed that SA+/ST−/mCRAMP^+/+^ and SA+/ST+/mCRAMP^−/−^ mice exhibited higher levels of phenylalanine, taurine, cadaverine, and carnitine at 48 hpi relative to the same treatments at 24 hpi. The concentration of phenylalanine in livers has previously been described to increase during periods of bacterial infection [61, 68]. This could be explained by an increase in the uptake of phenylalanine from the liver to produce acute phase proteins [61, 69]. Carnitine is an essential component of cellular metabolism, in charge of transporting activated long-chain fatty acids across the mitochondrial membrane for β-oxidation [70]. However, this metabolite has also been observed to modulate polymorphonuclear activities and the production of radical oxygen species [71]. Thus, all the alterations to the liver metabolome that we observed in the current study could be the result of higher colonization and damage induced by the pathogen. Moreover, as these changes were more prominent in mCRAMP deficient mice, the results are consistent with our hypothesis that the absence of mCRAMP increases susceptibility to *Salmonella* infection.

### The microbiota community balance is disrupted in mCRAMP-deficient mice infected with *Salmonella*

A principal goal of our study was to comparatively characterize the composition of the microbiota of mice receiving different treatments (i.e. SA+/ST−/mCRAMP^−/−^, SA+/ST+/mCRAMP^−/−^, SA+/ST−/mCRAMP^+/+^, SA+/ST+/mCRAMP^+/+^, SA−/ST−/mCRAMP^−/−^, SA−/ST+/mCRAMP^−/−^, SA−/ST−/mCRAMP^+/+^, and SA−/ST+/mCRAMP^+/+^). A comparison of SA−/ST−/mCRAMP^+/+^ and SA−/ST−/mCRAMP^−/−^ mice revealed no difference in diversity, but a subtle difference in the composition of the cecal microbiota between genotypes, which was attributed to the requisite segregated housing of the two genotypes. In contrast, substantive alterations in the composition and diversity of the microbiota were observed in ST+ mice, as has been reported previously [38]. In SA+/ST+/mCRAMP^−/−^ and SA+/ST+/mCRAMP^+/+^ mice, the bacterial community was dominated by the phylum, *Proteobacteria*, and the family, *Enterobacteriaceae*, as expected. This was attributable to the dysbiosis generated by streptomycin and the ensuing loss of colonization resistance, which primarily benefits members of the *Proteobacteria* including *Salmonella* [38]. The disproportionate increase of *Proteobacteria* is a hallmark of dysbiosis and epithelial injury [72]; higher levels of oxygen in the intestinal lumen are normally found in inflamed tissues [73] attributable to reactive oxygen species and oxidative burst established by neutrophils under infection [74]. Moreover, the diversity of bacteria observed in the cecal digesta of SA+/ST−/mCRAMP^−/−^ mice was reduced relative to SA+/ST−/mCRAMP^+/+^ mice, which we attributed to the higher levels of mucosal inflammation and an ensuing increase in oxygen that benefited *Proteobacteria* over obligate anaerobes [75]. It has previously been proposed that *Salmonella* induces inflammation to reduce colonization resistance, thereby allowing it to colonize the intestine and infect the host [76]. The higher abundance of *Enterobacteriaceae*, the increased degree of inflammation, and the severity of mucosal damage observed in the cecum of SA+/ST−/mCRAMP^−/−^ mice could be the result of direct or indirect changes, or both, associated with the mCRAMP deficiency.

The mechanisms of colonization resistance delivered by the enteric microbiota are essential to the well being of the host. When studying an intestinal pathology, the presence of a balanced microbiota is crucial to elucidate the complex interactions among the host, the autochthonous microbiota, and the pathogen [22]. Although the mechanisms of colonization resistance are enigmatic at present, the putative mechanisms have been divided into direct and indirect competition [77]. Indirect mechanisms of colonization resistance include production of short-chain fatty acids [78], modulation of cytokine release [28], and stimulation of cathelicidin secretion [3, 15] to avoid colonization. Additionally, the endogenous production of mCRAMP has also been observed to play an essential role in maintaining a balanced microbiota (i.e. a homeostasis) in the colon [21]. In the current study, we observed that alpha and beta diversity of the enteric microbiota did not differ between SA−/ST−/mCRAMP^−/−^ and SA−/ST−/mCRAMP^+/+^ mice. This indicates that the complex microbiota necessary to induce colonization resistance against *S.* Typhimurium [79] was conserved in mCRAMP deficient mice. However, in SA+/ST−/mCRAMP^−/−^ mice, a significantly lower Shannon diversity was detected in relation to SA+/ST−/mCRAMP^+/+^ mice indicating that a deficiency of mCRAMP may increase susceptibility to *Salmonella*, at least in part, due to the altered structure of the microbiota. Mice with a defined microbiota that were inoculated with *Akkermansia muciniphila* and *S.* Typhimurium showed a higher degree of inflammation [80]. This was attributed to the degradation of the mucus layer by the commensal *A. muciniphila*, which facilitated *Salmonella* attachment to the epithelium. It is noteworthy that in the current study, we observed that cecal digesta of SA+/ST−/mCRAMP^−/−^ mice possessed a higher abundance of the family *Akkermansiaceae* compared to SA+/ST−/mCRAMP^+/+^ mice. However, these bacteria were also present in SA+/ST−/mCRAMP^+/+^ mice, which suggests that they are not entirely responsible for the predisposition to infection that we observed in mCRAMP deficient mice. The administration of mCRAMP to the distal colon of mice has been previously demonstrated to attenuate dextran sulfate sodium-induced colitis by enhancing the mucus layer [81]. Additionally, expression of LL-37, the human homologue of mCRAMP, has been observed to enhance mucus production in airways [82]. Therefore, the inability of mice to produce cathelicidin may have enhanced salmonellosis by directly or indirectly disrupting the mucus layer. Additionally, this disruption of the mucus layer could have contributed to the proliferation of *Akkermansiaceae* in mCRAMP deficient mice aggravating the infection.

## Conclusion

We evaluated the progression of salmonellosis in mCRAMP^+/+^ and mCRAMP^−/−^ mice by characterizing histopathologic changes, host immune responses, and alterations to the liver metabolome and enteric microbiota. The higher upregulation of pro-inflammatory genes (*Ifnγ, Kc, Inos, Il1β*) and the higher concentration of immune proteins (MPO, KC) that we observed in SA+/ST−/mCRAMP^−/−^ and SA+/ST+/mCRAMP^−/−^ mice are clear indications of a higher susceptibility to salmonellosis when cathelicidin is absent. Moreover, the metabolic profiles of the livers of mice infected with *S*. Typhimurium differed between the mCRAMP^−/−^ and mCRAMP^+/+^ genotypes. This evidence demonstrated that mCRAMP deficient mice were predisposed to *Salmonella* infection both locally and systemically. Additionally, we demonstrated that mCRAMP plays a role in host response to infection by *S.* Typhimurium by altering the microbiota. In this regard, the higher abundance of *A. muciniphila* that we observed in SA+/ST−/mCRAMP^−/−^ and SA+/ST+/mCRAMP^–/−^ mice could be an indicator of a disruption to the mucus layer resulting from the absence of mCRAMP. It is noteworthy that the two C57BL/6 genotypes used in the current study were housed separately but adjacent to one another in the LeRDC small animal facility, and we observed a difference between the two in the structure of their intestinal microbiota albeit subtle. Thus, future research to elucidate mechanisms of mCRAMP function should take steps to address this (e.g. select breeding of mCRAMP^–/−^ and mCRAMP^+/+^ mice). Furthermore, research could involve studies in which mCRAMP is delivered to sites of intestinal inflammation incited by *S.* Typhimurium in mCRAMP^–/−^ mice.

## Materials and methods

### Ethics statement

The study was carried out in strict accordance with the recommendations established in the Canadian Council on Animal Care Guidelines. The project was reviewed and approved by the Lethbridge Research and Development Centre (LeRDC) Animal Care Committee (Animal Use Protocol Review 1729), and the LeRDC Biosafety and Biosecurity Committee before commencement of the research.

### Experimental design

The experiment was designed as a two (± mCRAMP) by two (± *Salmonella*) by two (± streptomycin) by two (sample time) factorial experiment, arranged as a completely randomized design (CRD). Three replicates were conducted on separate occasions to ensure independence; 16 mice were used per replicate with a total of 48 mice used for the entire experiment (Additional file 1: Fig. S7).

### Animal maintenance

Specific-pathogen-free agnotobiotic C57BL/6 mice and mCRAMP knockout mice were purchased from The Jackson Laboratory (Bar Harbor, ME) and used to establish breeding colonies at LeRDC. The breeding colony of mCRAMP^−/−^ mice was maintained in an isolator (CBClean, Madison, WI), whereas mCRAMP^+/+^ mice were maintained in conventional cages in an adjacent animal room. Individual mCRAMP^−/−^ and mCRAMP^+/+^ mice at 6-weeks-of-age were transferred to individually ventilated cages (IVCs) connected to a HEPA filter unit (Techniplast, Montreal, QC) operated in containment mode. Mice were individually housed, but could see each other through the transparent plastic cages. Mice were acclimatized to the IVCs for 7 days before commencement of the experiment. Mice were maintained on a Prolab RMH 3500, Autoclavable 5P04 diet (LabDiet, St. Louis, MO), and they were permitted to eat and drink ad libitum.

### Inoculation and streptomycin administration

Mice were intragastrically administered either 100 µL of streptomycin (20 mg/µL) or 100 µL of water alone using a 22G, rigid gavage needle (Western Drug Distribution Centre Ltd, Edmonton, AB). Twenty-four h after administration of the antibiotic or water, mice were orally inoculated with a 100 µL of *S. enterica* serovar Typhimurium DT104 (strain SA970934) [41] or Columbia Broth (CB) alone (VWR, Mississauga, ON) as described above for administration of streptomycin. To produce inoculum, the bacterium was grown aerobically on MacConkey’s agar (MA) (Difco BD, Mississauga, ON) at 37 °C for 24 h. Biomass was removed from the surface of the agar and transferred into CB. Cultures were maintained for 180 to 210 min at 37 °C with shaking at 150 rpm, until an optical density (600 nm) of 1.2 or greater was obtained. Cultures were centrifuged at 4000×*g* for 15 min, supernatants were removed to a volume of 15 mL, and the optical density was adjusted to a target of 3.0 x 10^9^ cells/mL. To ascertain *S*. Typhimurium cell densities, the suspension was diluted in a tenfold dilution series, 100 µL of each dilution was spread in duplicate onto MA, cultures were incubated aerobically at 37 °C, and colonies were counted at the dilution yielding 30 to 300 colony forming units after 24 h.

### Animal health status and tissue collection

Mice were monitored daily for changes in health status, which included evidence of diarrhea, altered food consumption, and behavioral changes (e.g. restless and depressed). Twenty-four and 48 hpi, animals were anaesthetized with isoflurane (Western Drug Distribution Centre Ltd). Under anesthesia, blood was collected intracardially, and animals were humanly euthanized by cervical dislocation. Immediately after death, a laparotomy was completed, and viscera was exposed. The liver, spleen, and intestine were aseptically collected.

### Histopathology

Tissue samples from the duodenum, jejunum, ileum, cecum, and proximal and distal colon were placed in TrueFlow Macrosette cassettes (Tissue Path; Thermo Fisher Scientific, Edmonton, AB), and submerged in 10% neutral buffered formalin (Surgipath Canada, Inc., Winnipeg, MB). Samples were dehydrated using a tissue processor (Leica TP 1020, Leica Biosystems, Location), and embedded in paraffin (Fisherfinest™ Histoplast PE; Thermo Fisher Scientific) using a Shandon Histocentre 3 (Thermo Fisher Scientific). Using a Shandon Finesse 325 microtome (Thermo Fisher Scientific), 5-μm-thick sections were transferred to positively charged slides (Fisherbrand Superfrost™ Plus Gold; Thermo Fisher Scientific) and allowed to dry prior to being deparaffinized with xylene. Slides were rehydrated in ethanol and stained with H&E using a standard protocol. Histopathologic changes were scored in a blinded fashion as to treatment by a board-certified pathologist (V.F.B). The scoring system used was developed from Boyer et al. [83], Garner et al. [84], Koelink et al. [85], and Erben et al. [86]. The tissues were scored for severity of inflammatory cell infiltrate (1–4), infiltration extent (1–3), epithelial hyperplasia (1–5), goblet cell loss (1–4), cryptitis (2–3), epithelial injury (0–4), irregular crypts (4–5), and crypt loss (0–2). Scores were combined across all categories to obtain the total histopathologic score (maximum score of 30) (Additional file 1: Table S3).

### Bacterial genomic DNA extraction

For quantification of *S*. Typhimurium associated with intestinal and liver tissues by quantitative (q)PCR, DNA was extracted from the ileum, cecum, proximal colon, and liver using the Qiagen Blood and Tissue kit (Qiagen Inc., Toronto, ON) following the gram positive protocol as recommended by the manufacturer. For quantification of *S*. Typhimurium and characterization of bacterial communities within digesta, DNA was extracted from cecal digesta using the Qiagen Fast DNA Stool Mini Kit (Qiagen Inc.) according to the manufacturer’s protocol. A bead beating step using 5.0-mm-diam stainless steel beads and a TissueLyser LT (Qiagen Inc.) at 30 Hz was added to ensure efficient release of genomic DNA from both Gram positive and Gram Negative bacteria; the bead beating step was conducted three times (30 s duration).

### Quantification of *Salmonella*

Duplicate PCR reactions were prepared. Each reaction contained 10 μL QuantiTect SYBR Green Mastermix (Qiagen Inc.), 0.5 μM of the forward and reverse primers (IDT, San Diego, CA), 2 μg of BSA (Promega, Madison, WI), 2 μL of DNA, and 4 μL of nuclease free water (Qiagen Inc.). The primers used were F-(Sal) and R-(Sal) [87]. Data was collected using an Mx3005p Realtime PCR instrument (Agilent Technologies Canada Inc. Mississauga, ON). Cycle conditions were 95 °C for 15 min, followed by 40 cycles of 94 °C for 15 s, 64 °C for 30 s, and 72 °C for 30 s. A standard curve was prepared with serial dilutions of genomic DNA (2.6 x 10^6^ copies/g) extracted from pure cultures of the pathogen. A dissociation curve (55–95 °C) was included with each run to verify amplicon specificity. All reactions were run in duplicate, and average Ct values were calculated.

### Quantification of immune genes

Within 15 min of death, samples from the ileum and cecum (≈ 0.5 x 0.5 cm) were placed in RNAprotect^®^ (Qiagen Inc.) and stored at − 20 °C until processed. RNA was extracted from the samples using an RNeasy Mini Kit (Qiagen Inc.) with a DNase step added to eliminate residual genomic DNA (Qiagen Inc.). RNA quantity and quality was determined using Bioanalyzer 2100 (Agilent Technologies Canada Inc., Mississauga, ON) and 1000 ng of RNA was transcribed to cDNA (Qiagen Inc.). Evaluation of innate immune defenses included expression of host defense peptide (*RegIIIγ*, *mCramp*), pattern recognition receptor (*Tlr2, Tlr4, Tlr5*), phagocytes (*Il18, iNOS, Il1β*), epithelial barrier (*Kc, Zo1, Occludin, Muc2*), T-helper (*Ifnγ, Il22, Il4*) and T-regulatory (*Il10, Tgfβ*) responses. Reactions were run in a 384-well plate containing 5.0 μl QuantiTect SYBR Green Master Mix (Qiagen Inc.), 0.5 μL of each primer (10 μM), 3.0 μL of RNase-free water, and 1.0 μL of cDNA. Quantitative PCR was performed in ABI 7900HT thermocycler (Applied Biosystems, Carlsbad, CA). Cycle conditions consisted of 95 °C for 15 min, followed by 40 cycles of 95 °C for 15 s, 58–62 °C for 30 s, and 72 °C for 30 s. A dissociation curve (55-95 °C) was included. All reactions were run in triplicate. Average Ct values were used to calculate gene expression normalized to Peptidylprolyl isomerase A (*Ppia*), hypoxanthine–guanine phosphoribosyltransferase (*Hprt*), and beta-glucuronidase (*Gusβ*) reference genes. These genes were selected using the geNorm algorithm in qbase + (Biogazelle, Zwijnaarde, Belgium) based on stability among samples.

### Quantification of immune peptides and proteins

Blood collected by intracardiac puncture was directly transferred to a BD Microtainer^®^ SST tubes (BD, Franklin Lake, NJ) and processed according to the manufacturer’s directions. Serum was aliquoted into two 2 mL cryovials and stored at − 80 °C until processed. The left lateral lobe of the liver and a section of ileum were snap frozen in liquid nitrogen, and stored at − 80 °C until processing. To homogenize tissues, samples from each mouse were suspended in immunoprecipitation buffer at a ratio of 1:5 (w/v). The buffer was adapted from Burgos-Ramos et al. [88], and consisted of 50 mM of NaH_2_PO_4_, 100 mM Na_2_PO_4_, 0.1% sodium dodecyl sulfate, 0.5% NaCl, 1% Triton X-100, and 5 mg/mL sodium deoxycholate, with the addition of a 1% Protease Inhibitor Cocktail (Sigma Aldrich, St. Louis, MO). Frozen samples were immediately homogenized using a Tissue-Tearor^®^ model 398 homogenizer (Biospec Products, Bartlesville, OK), and centrifuged at 14,000×*g* for 30 min at 4 °C. Supernatants were collected and stored at − 80 °C until analysed. Cytokine concentrations were measured by enzyme-linked immunosorbent assay (ELISA) using freshly thawed serum and tissue homogenates. The pro-inflammatory chemokine, neutrophil chemoattractant CXCL1/KC was measured. In addition, neutrophil enzyme myeloperoxidase (MPO) and murine-cathelicidin related antimicrobial peptide (mCRAMP) were measured. Proteins were quantified using mouse DuoSet ELISA Development kits according to the manufacturer’s protocols (R&D Systems, Minneapolis, MN). ELISAs were performed using 96-well high-binding half area microplates (Greiner Bio-One, Frickenhausen, Germany). Quantification of mCRAMP was carried out using a mouse Camp ELISA Kit following the manufacturer’s instructions (Aviva Systems Biology, San Diego, CA). Optical densities of the reactions were determined at a wavelength of 450 nm on a Synergy HT multi-detection microplate reader (BioTek Instruments Inc, Winooski, VT) with Gen5 analysis software (BioTek Instruments Inc., Winooski, VT).

### Flow cytometric analysis of splenic immune cell populations

Excised spleens were placed on a sterile 70 μm cell strainer and crushed using a 3 mL syringe plunger with RMPI-1640 cell medium (Sigma Aldrich) containing 10% fetal bovine serum (FBS) (Fisher Scientific). Splenic cells were pelleted by centrifugation and red blood cells were lysed using one times RBC Lysis Buffer (Invitrogen, Carlsbad CA). Cells were subjected to viability staining using Ghost Dye™ Red 780 (Tonbo Biosciences, San Diego, CA) followed by an Fc blocking step with 10% heat-inactivated FBS. Samples were divided into three separate panels with 1 × 10^6^ cells each in an effort to maximize the amount of cell populations analyzed. The first panel included anti-CD3-PerCP and anti-IL23R-PE (R&D Systems) with anti-CD4 PE-Cy7, anti-CD8-FITC and anti-TCR-β-APC (Tonbo Biosciences). The second panel utilized anti-CD3-PerCP, anti-CD161-PE-Cy7 (Invitrogen), anti-CD11b-FITC, and anti-CD11c-PE (Tonbo Biosciences). The third panel included anti-CD45-PE-Cy7, anti-CD11b-FITC, and anti-Ly-6G-APC (Tonbo Biosciences) with anti-CD18-PE and anti-Ly-6C-PerCP (BioLegend, San Diego, CA). Cells were subsequently fixed with Intracellular Fixation and Permeabilization Buffer (Invitrogen) according to the manufacturer’s instructions, and the second panel subset was further stained intracellularly with anti-CD68-APC or its corresponding IgG2aκ isotype control (BioLegend). Samples were acquired on a BD FACSCanto II (BD Biosciences, San Jose, CA) flow cytometer equipped with blue 488 nm and red 633 nm lasers, and cell populations were analyzed using FlowJo v10 (BD Biosciences, Ashland, OR).

### Analysis of bacterial communities

DNA extracted for community analysis was quantified with a Qubit (Thermo Fisher Scientific). Library preparation, next generation sequencing and quality control was performed by McGill University and Genome Quebec Innovation Centre (Montreal, QC). Bacterial 16S rRNA libraries were amplified with the Illumina index adaptor primers 341F (5′-CCTACGGGNGGCWGCAG-3′) and 805R (5′-GACTACHVGGGTATCTAATCC-3′), and run on an Illumina MiSeq platform. QIIME2 [89] was used to classify bacterial reads from digesta communities. Raw reads were denoised with DADA 2 [90], and representative sequences and ASVs were generated. A phylogenetic tree of ASVs sequences was generated, and the taxonomy of each ASV was identified by using a machine learning classifier pre-trained with the reference SILVA 132 database (silva-132-99-341-806-nb-classifier.qza). Alpha diversity metrics including number of taxa observed, Pielou’s evenness, Shannon’s index of diversity, and the Faith’s index were calculated. The phyloseq package (version 1.28.0) of R version 3.6.1 was used to evaluate beta-diversity with a principal coordinate analysis (PCoA) of the calculated unweighted and weighted UniFrac distances, generating ordination plots. Detection of differential abundance between tissues was done with Gneiss in QIIME2 [91].

### Metabolomics

Samples from the right medial lobe of the liver were collected, fast frozen in liquid nitrogen and stored at -80 °C. In order to extract water-soluble metabolites, samples were thawed on ice, 100 mg of tissue was mixed with 4 mL/g of methanol and 1.6 mL/g of deionized H_2_O, and vortexed (high setting) until thoroughly mixed. Samples were then homogenized with a TissueLyser LT (Qiagen Inc.) and 5-mm-diam stainless steel beads for 5 min at 50 Hz, vortexed for 1 min, and process repeated two times. Chloroform (4 mL/g) and deionized H_2_O (4 mL/g) were added to the homogenate and mixed thoroughly by vortexing. Samples were kept at 4 °C for 15 min, centrifuged at 4 °C for 15 min at 1000×*g*, the supernatant (600 µL) transferred to a new tube, and the tube with the lid removed was maintained in an operating fume hood for 4 days to allow the solvents to evaporate. The remaining pellet was resuspended in 480 µL of metabolomics buffer (0.125 M KH_2_PO_4_, 0.5 M K_2_HPO_4_, 0.00375 M NaN_3_, and 0.375 M KF; pH 7.4). A 120 µL aliquot of deuterium oxide containing 0.05% v/v trimethylsilylpropanoic acid (TMSP) was added to each sample (final total volume of 600 μL); TMPS was used as a chemical shift reference for 1H-NMR spectroscopy. A 550 μL aliquot was then loaded into a 5 mm NMR tube and run on a 700 MHz Bruker Avance III HD spectrometer (Bruker, ON, Canada) for spectral collection. Data acquisition and processing were followed as previously described [92].

### Statistical analysis

Statistical analyses for gene and protein expression, histopathologic measurements, *S.* Typhimurium quantification, cell populations and immune protein quantification were performed using Statistical Analysis Software (SAS Institute Inc. Cary, NC). With the exception of histopathologic data, normality was confirmed, and data was analyzed using the MIXED procedure of SAS. In the event of a main effect (P ≤ 0.050), the least squares means test was used to compare treatments within factors. Histopathologic measurement data was analyzed using the pairwise Fisher’s exact test in SAS. Data is represented by mean ± standard error of the mean (SEM).

Metabolomics NMR spectra were exported to MATLAB (Math Works, MA, USA) where they underwent spectral peak alignment and binning using Recursive Segment Wise Peak Alignment [93] and Dynamic Adaptive Binning [94], respectively. After these analyses the dataset was then normalized to the total metabolome, excluding the region containing the water peak, and pareto scaled. The MetaboanalystR package was used to perform univariate and multivariate statistics including calculation of fold changes of specific metabolites, principal component analysis, heat map creation, and hierarchical clustering analysis [95]. These tests were carried out using the bins identified as significant by univariate tests in order to observe group separation. Univariate measures included the t-test and the Mann–Whitney U test. Both tests determine if there is a significant difference between the means of the two groups; however, the t-test and the Mann–Whitney U test are applied in the case where the data is normally distributed (parametric) or not, respectively. The test for data normality was carried out using a decision tree algorithm as described by Goodpaster et al. [96]. All p-values obtained from analysis were Bonferroni-Holm corrected for multiple comparisons. Metabolites were then identified using Chenomx 8.2 NMR Suite (Chenomx Inc., AB, Canada).

## Supplementary information

**Additional file 1.** Additional figures and tables.

## Data Availability

The datasets generated during and/or analyzed during the current study are available from the corresponding authors on reasonable request.

## Abbreviations

hpi: Hours post-inoculation
mCRAMP: Murine cathelicidin-related antimicrobial peptide
mCRAMP^−/−^: Mice deficient in mCRAMP
mCRAMP^+/+^: Wild type mice
SA+: Mice inoculated with *S.* Typhimurium
SA−: Mice not inoculated with *S.* Typhimurium
ST+: Mice pretreated with streptomycin
ST−: Mice not pretreated with streptomycin
LL37/hCAP18: Human cationic antimicrobial peptide
FPR: Formyl peptide receptor
TLR: Toll-like receptor
KC: Keratinocyte-derived chemokine
iNOS: Inducible nitric oxide synthase
IL: Interleukin
RegIII: Regenerating islet-derived protein
IFN: Inteferon
MUC: Membrane bound mucin
ZO: Zonula occludens
TGF: Tumor growth factor
MPO: Myeloperoxidase
NK: Natural killer
Th: T helper lymphocyte
NMR: Nuclear magnetic resonance
NGS: Next generation sequencing
ASV: Amplicon sequence variants
HOCL: Hypochlorus acid
CRD: Complete randomized designed
LeRDC: Lethbridge Research and Development Centre
IVCs: Individual ventilated cages
CB: Columbia Broth
H&E: Hematoxylin and eosin
HPRT: Hypoxanthine-guanine phosphoribosyltransferase
Gus: Glucuronidase
ELISA: Enzyme-linked immunosorbent assay
PCoA: Principal coordinate analysis
PCA: Principal component analysis
TMSP: Trimethylsilylpropanoic acid
SEM: Standard error of the mean
