# Supplementary material for: The absence of murine cathelicidin-related antimicrobial peptide impacts host responses enhancing Salmonella enterica serovar Typhimurium infection
Source: Gut Pathog. 2020 Nov 13;12:53. doi: 10.1186/s13099-020-00386-1 (PMC7666523; doi:10.1186/s13099-020-00386-1)
Supplement: Supplementary file 1 — Additional file 1. Additional figures and tables. [file 13099_2020_386_MOESM1_ESM.pdf]

Supplemental material

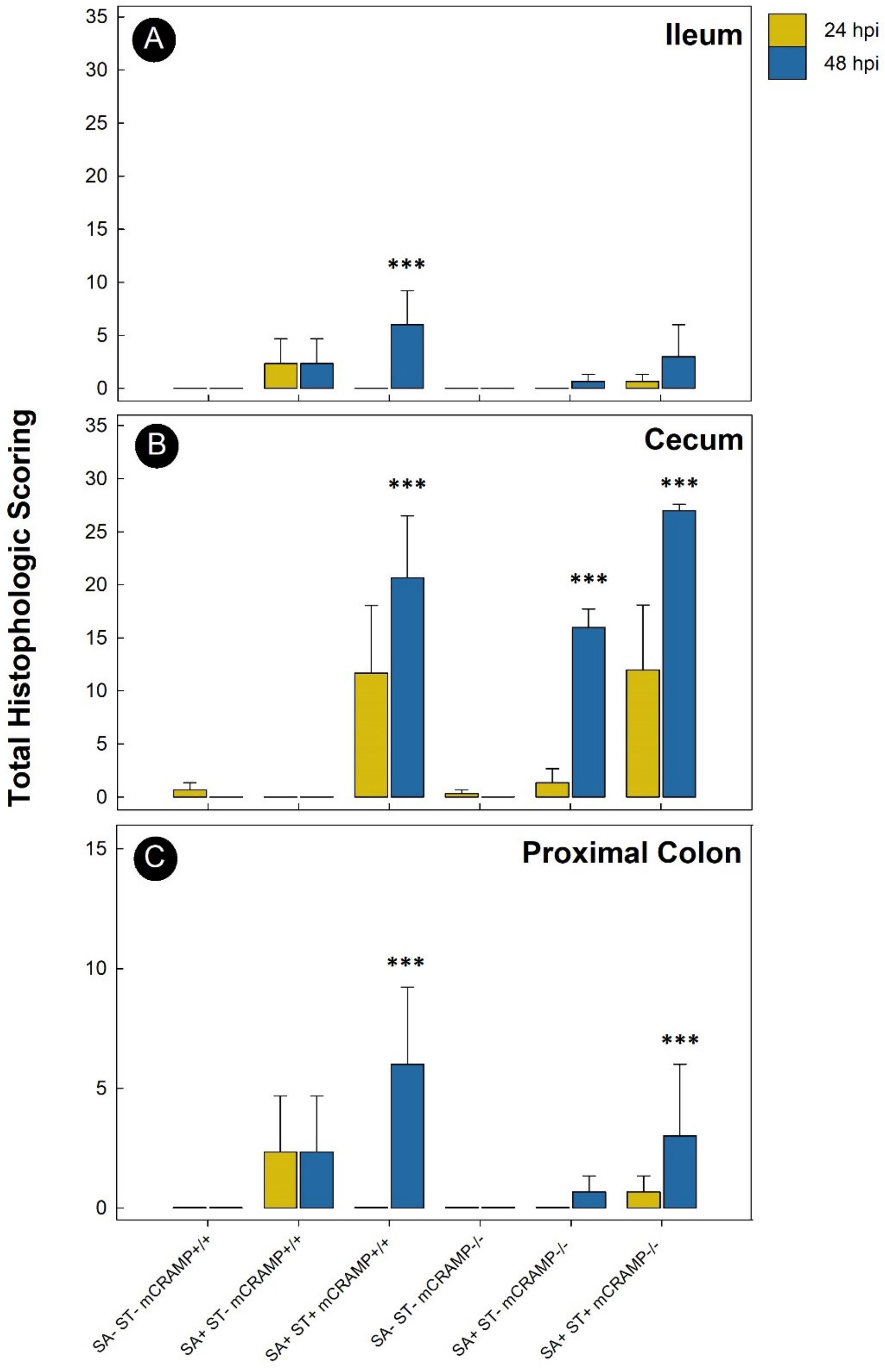

Fig S1 Total histopathologic scores in mCRAMP$^{-/-}$ and mCRAMP$^{+/+}$ mice that were inoculated with *Salmonella enterica* Typhimurium (SA+) or medium alone (SA-), and pre-treated with streptomycin (ST+) or water alone (ST-) at 24 vs 48 hours post-inoculation. (A) Ileum; (B) Cecum; (C) Proximal Colon. Vertical lines associated with markers are standard errors of the mean. Histogram bars with asterisks indicate that treatments differ (***P<0.001).

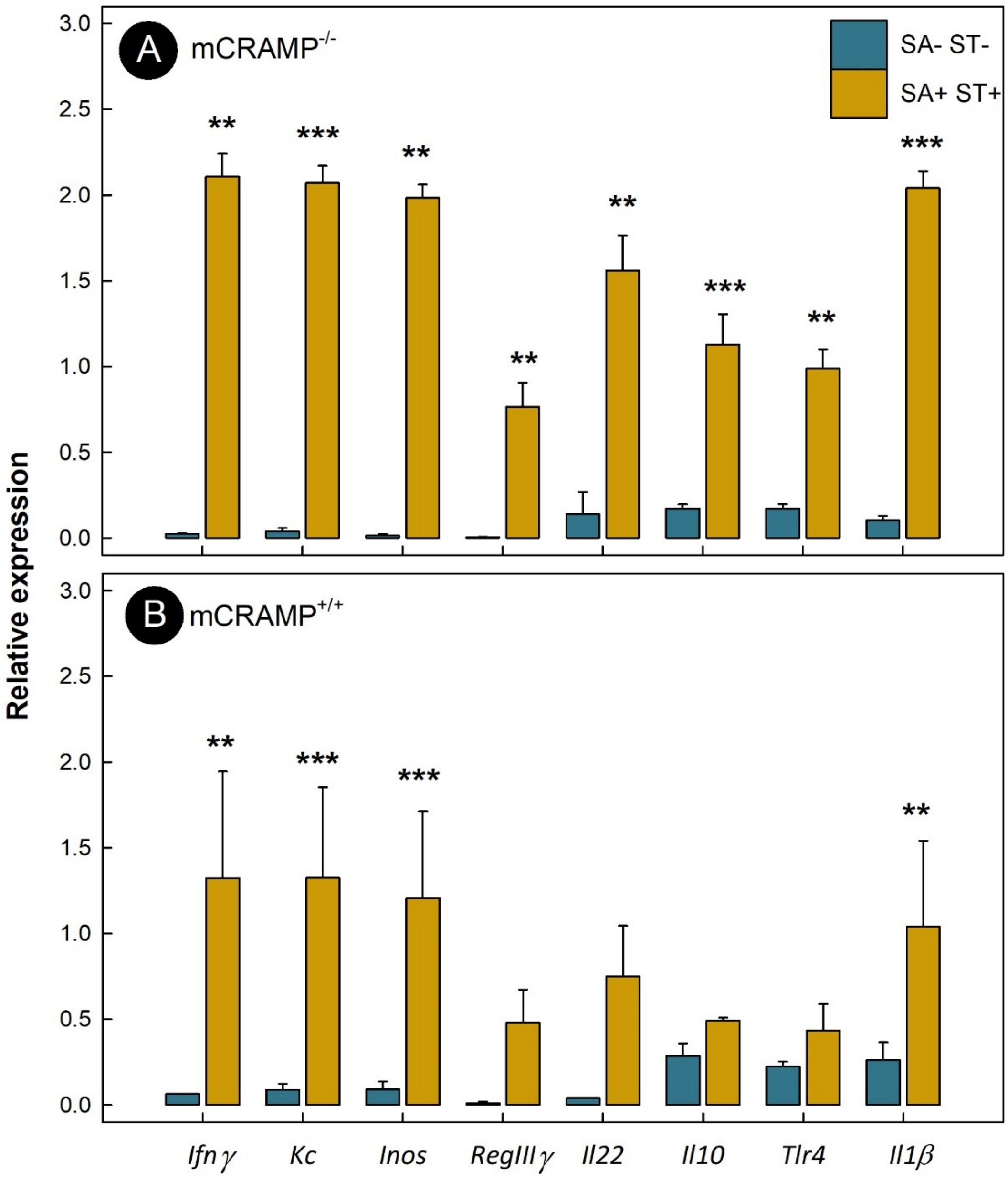

Fig S2 Relative gene expression in cecum of mCRAMP$^{+/+}$ and mCRAMP$^{-/-}$ mice that were inoculated with *Salmonella enterica* Typhimurium (SA+) or medium alone (SA-) and pretreated with streptomycin (ST+) or water alone (ST-) at 48 hours post-inoculation. (A) mCRAMP$^{-/-}$ (B) mCRAMP$^{+/+}$. Vertical lines associated with markers are standard errors of the mean. Histogram bars with asterisks indicate that treatments differ (*P<0.050, **P<0.010).

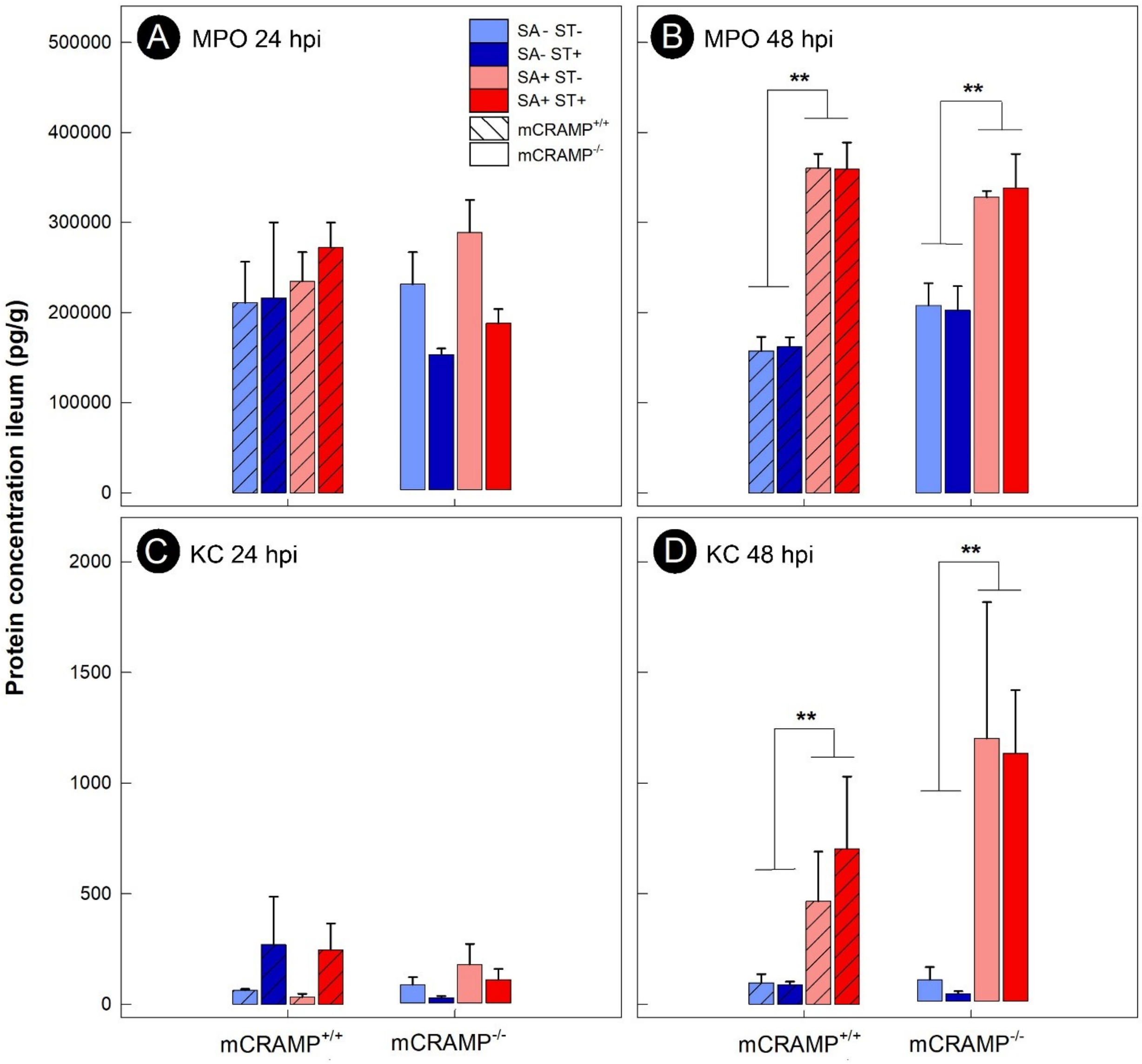

Fig S3 Protein concentrations of MPO and KC in the ileum of mCRAMP$^{+/+}$ and mCRAMP$^{-/-}$ mice that were orally inoculated with *Salmonella enterica* Typhimurium (SA+) or medium alone (SA-), and pretreated with streptomycin (ST+) or water alone (ST-). (A) MPO at 24 hours post-inoculation (hpi); (B) MPO at 48 hpi; (C) KC at 24 hpi; (D) KC at 48 hpi. Vertical lines associated with markers are standard errors of the mean. Histogram bars with asterisks indicate that treatments differ (*P<0.050, **P<0.010, ***P<0.001).

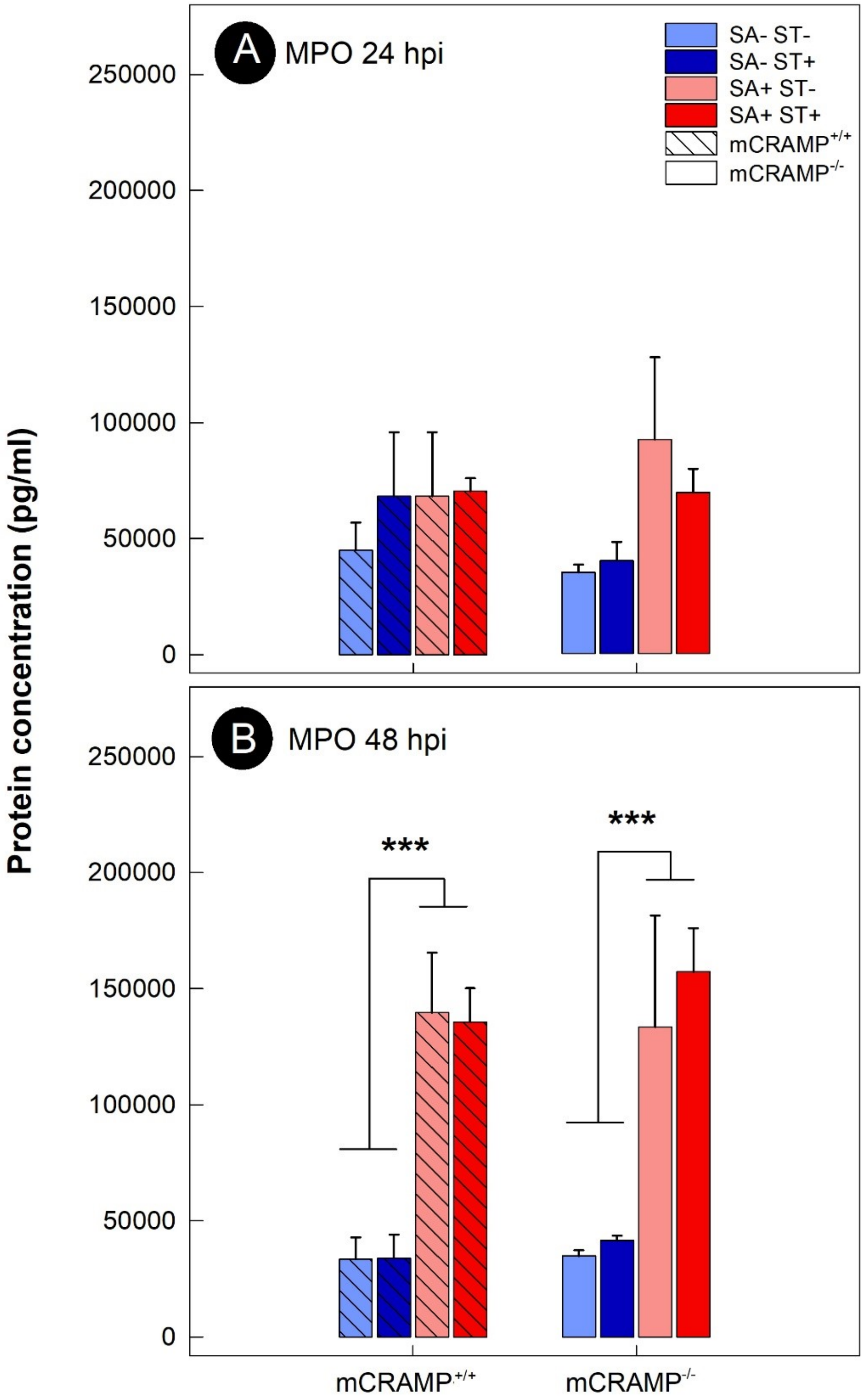

Fig S4 Protein concentrations of MPO in the serum of mCRAMP$^{+/+}$ and mCRAMP$^{-/-}$ mice that were orally inoculated with *Salmonella enterica* Typhimurium (SA+) or medium alone (SA-), and pretreated with streptomycin (ST+) or water alone (ST-). (A) MPO at 24 hours post-inoculation (hpi); (B) MPO at 48 hpi. Vertical lines associated with markers are standard errors of the mean. Histogram bars with asterisks indicate that treatments differ (*P<0.050, **P<0.010, ***P<0.001).

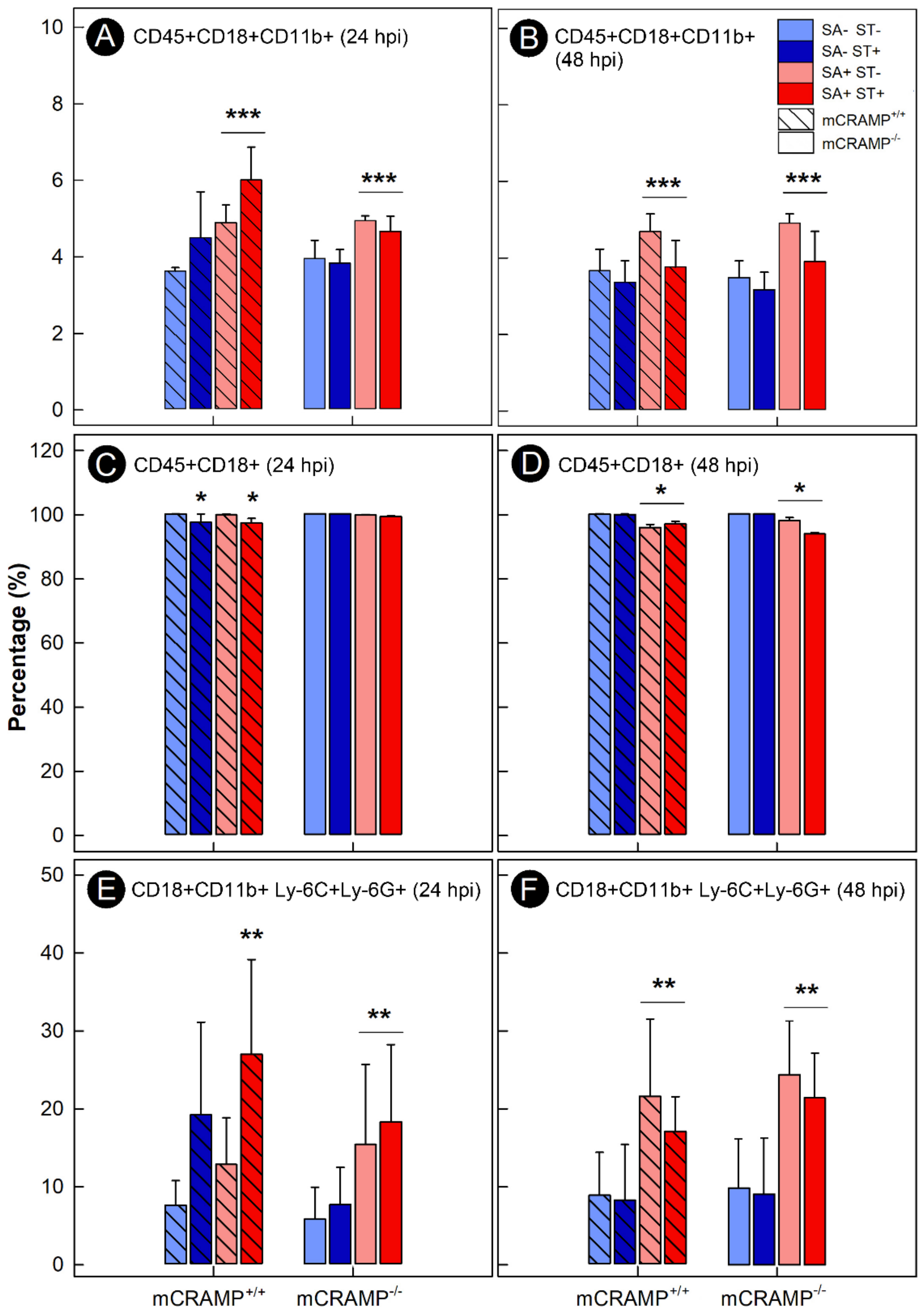

FIG S5 Percentage of splenic immune cell populations in mCRAMP$^{-/-}$ and mCRAMP$^{+/+}$ mice, that were orally inoculated with *Salmonella enterica* Typhimurium (SA+) or medium alone (SA-), and pretreated with streptomycin (ST+) or water alone (ST-). (A) CD45$^+$CD18$^+$CD11b$^+$ leukocytes at 24 hours post-inoculation (hpi); (B) CD45$^+$CD18$^+$CD11b$^+$ leukocytes at 48 hpi; (C) CD45$^+$CD18$^+$ leukocytes at 24 hpi; (D) CD45$^+$CD18$^+$ leukocytes at 48 hpi; (E) CD18$^+$CD11b$^+$ Ly-6C$^+$Ly-6G$^+$ neutrophils at 24 hpi; (F) CD18$^+$CD11b$^+$ Ly-6C$^+$Ly-6G$^+$ neutrophils at 48 hpi. Vertical lines associated with markers are standard errors of the mean. Histogram bars with asterisks indicate that treatments differ (*P<0.050, **P<0.010, ***P<0.001).

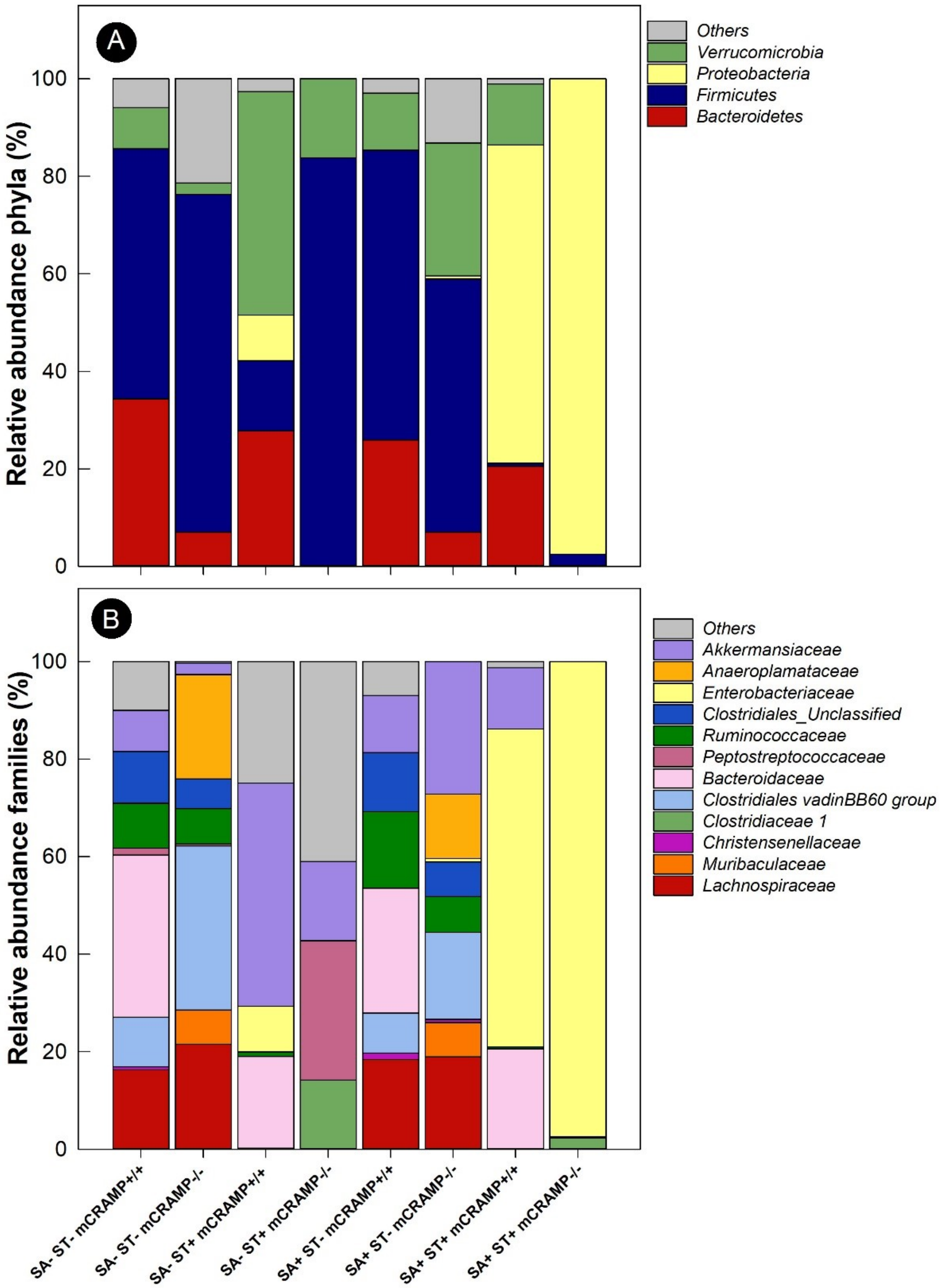

FIG S6 Relative abundance (%) of bacterial phyla and families in cecum digesta of mCRAMP$^{+/+}$ and mCRAMP$^{-/-}$mice that were orally inoculated with *Salmonella enterica* Typhimurium (SA+) or medium alone (SA-), and pretreated with streptomycin (ST+) or water (ST-) alone at 48 hours post-inoculation. (A) Phyla; (B) Families.

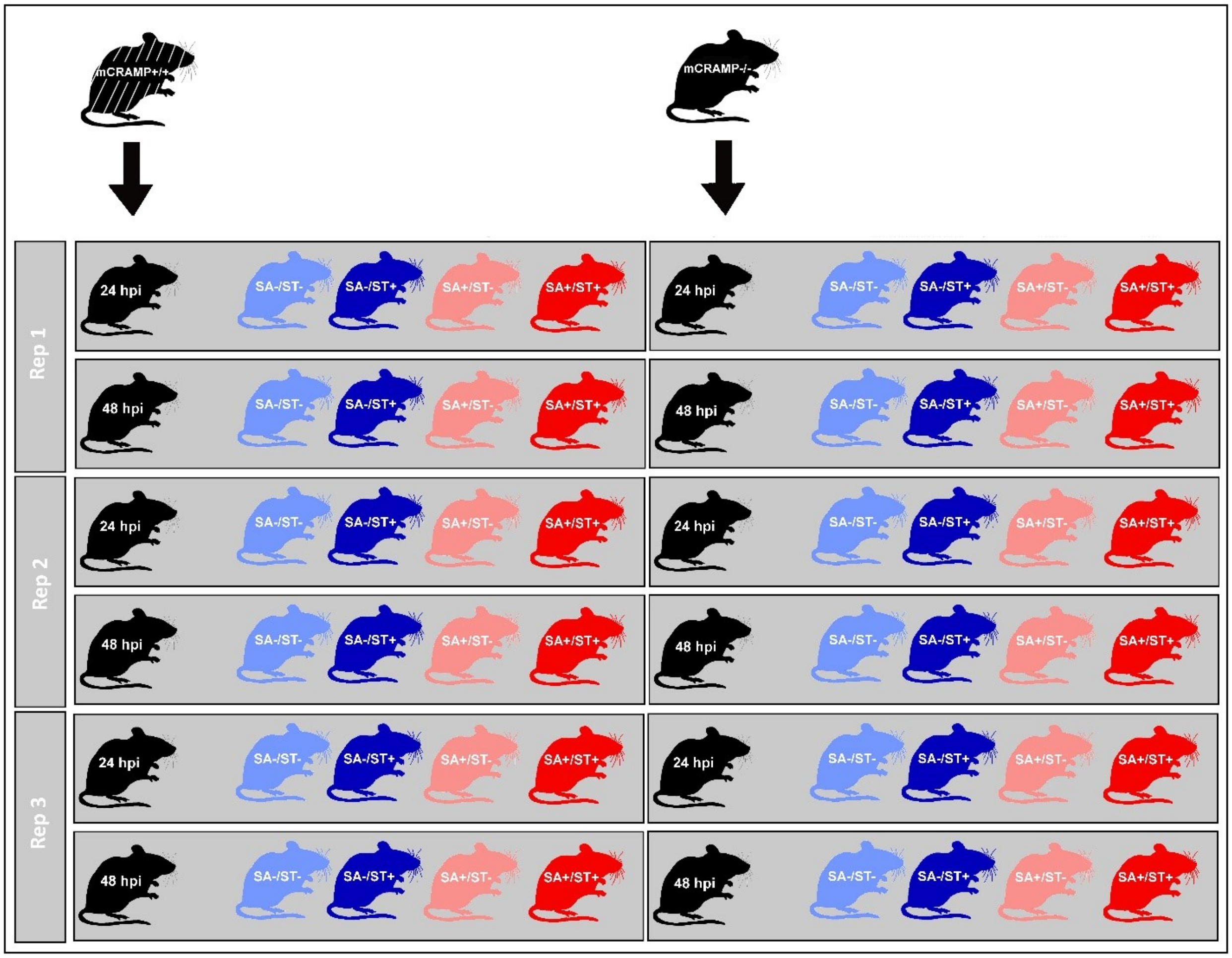

FIG S7 Schematic representation of the experimental design. The experiment was arranged as a two (± mCRAMP) by two (± *Salmonella*) by two (± streptomycin) by two (sample time) factorial experiment.The experiment was run on three separate occasions, and comprised 48 mice in total. The figure represents only one replicate.

Table S1 Splenic immune cell populations at 48 hours post-inoculation.

**Cell phenotype**	**mCRAMP$^{+/+}$**								**mCRAMP$^{-/-}$**							
	**ST- SA-**		**ST+ SA-**		**ST- SA+**		**ST+ SA+**		**ST- SA-**		**ST+ SA-**		**ST- SA+**		**ST+ SA+**	
	**Mean**	**SEM**	**Mean**	**SEM**	**Mean**	**SEM**	**Mean**	**SEM**	**Mean**	**SEM**	**Mean**	**SEM**	**Mean**	**SEM**	**Mean**	**SEM**
CD3$^+$ T cells (% of live lymphocytes)	1.21	0.2	1.45	0.39	1.59	0.28	1.55	0.36	0.94	0.3	0.76	0.06	1.16	0.14	0.97	0.09
CD4$^+$ T helper cells (% of CD3$^+$ cells)	38.0	6.15	44.3	1.05	43.7	5.08	52.0	8.47	42.36	5.24	49.33	9.70	45.96	7.87	53.13	5.34
CD8$^+$ cytotoxic T cells (% of CD3$^+$ cells)	3.4	1.20	2.42	0.55	4.10	1.44	4.20	0.92	4.88	2.57	4.04	1.60	5.06	1.91	5.43	1.76
CD3$^+$CD4$^+$TCR$^+$ γδ T cells (% of CD4$^+$ cells)	90.9	0.68	87.9	1.96	94.0	2.70	94.6	0.77	89.26	2.23	89.90	1.70	91.56	3.73	93.93	0.56
IL-23R$^+$ γδ T cells (% of CD4$^+$TCR$^+$ cells)	43.5	12.7	32.0	15.1	34.3	16.7	24.2	10.1	34.6	13.1	34.9	7.3	29.73	10.8	17.3	8.27
CD3$^-$CD161$^+$ NK cells (% of live CD3$^-$ cells)	4.00	0.30	3.06	0.18	3.16	0.34	2.46	0.14	3.72	0.18	3.56	0.11	3.48	0.49	3.03	0.34
CD3$^-$CD11b$^+$CD11c$^-$ monocytes (% of live CD3$^-$CD161$^-$ cells)	0.10	0.07	0.86	0.64	0.21	0.11	0.35	0.30	0.11	0.08	0.07	0.05	0.94	0.89	0.66	0.56
CD3$^-$CD11b$^+$CD11c$^+$ mDC (% of live CD3$^-$CD161$^-$ cells)	0.17	0.12	0.20	0.09	0.13	0.08	0.10	0.07	0.14	0.11	0.16	0.13	0.10	0.05	0.10	0.06
CD3$^-$CD11b$^-$CD11c$^+$ pDC (% of live CD3$^-$CD161$^-$ cells)	0.20	0.03	0.19	0.01	0.26	0.04	0.29	0.11	6.10	5.95	0.15	0.04	0.27	0.13	0.22	0.03
CD68$^+$ macrophages (% of CD11b$^+$CD11c$^+$ cells)	0.16	0.12	0.10	0.07	0.13	0.08	0.20	0.09	0.14	0.10	0.16	0.13	0.09	0.05	0.10	0.06

pDC, plasmacytoid dendritic cells; mDC, myeloid dendritic cells.

Table S2 P values for Alpha and Beta Diversity measurements.

**Treatments**		**Alpha Diversity**			**Beta Diversity**
**mCRAMP$^{+/+}$ vs mCRAMP$^{-/-}$**		**Pielou's evenness**	**Faith's index**	**ASVs counts**	**UniFrac weighted**
24 hpi	SA- ST-	0.563	0.248	0.248	0.201
	SA- ST+	0.512	0.055	0.126	0.511
	SA+ ST-	0.512	0.017	0.049	0.003
	SA+ ST+	0.049	0.126	0.275	0.306
48 hpi	SA- ST-	0.512	0.126	0.126	0.100
	SA- ST+	0.827	0.055	0.268	0.222
	SA+ ST-	0.563	0.017	0.563	0.003
	SA+ ST+	0.512	0.827	0.512	0.587

Table S3 Histopathologic scoring system

Score	Inflammatory cell infiltrate		Epithelial changes			Mucosal architecture	
	**Severity**	**Extent**	**Hyperplasia**	**Goblet cell loss**	**Epithelial injury**	**Irregular crypt***	**Crypt loss**
0	normal	normal	normal	normal	none	_	normal
1	Minimal: <10%	Mucosal	Minimal: <25%	Minimal <20%	rare (i.e. < 10 surface epithelial cells shedding)	_	decrease of <10%
2	Mild: 10-25%; scattered neutrophils	Mucosal and submucosal	Mild: 25-35%	Mild 21-35%	mild (i.e. focal epithelial erosions; 11-50 surface epithelial cells shedding)	_	decrease of ≥10%
3	Moderate: 26-50%	Mucosal, submucosal and transmural	Moderate: 36-50%; mitoses in middle/upper third of crypt epithelium, distant from crypt base	Moderate 36-50%	moderate (i.e. multi focal surface epithelium erosion; < 50 of surface epithelial cells shedding)	_	
4	Marked: >51%; dense infiltrate	_	Marked: >51%; mitoses in upper third of crypt epithelium, distant from crypt base	Marked >50%	severe (i.e. multi focal to coalescing surface epithelium erosions; >50 of surface epithelial cells shedding).	Yes	
5	_	_				Yes	

*Non-parallel crypts, variable crypt diameters, bifurcation and branched crypts
